# Repertoire-scale determination of class II MHC peptide binding via yeast display improves antigen prediction

**DOI:** 10.1038/s41467-020-18204-2

**Published:** 2020-09-04

**Authors:** C. Garrett Rappazzo, Brooke D. Huisman, Michael E. Birnbaum

**Affiliations:** 1 Koch Institute for Integrative Cancer Research, Cambridge, MA USA; 2 Department of Biological Engineering, Massachusetts Institute of Technology, Cambridge, MA USA; 3 Ragon Institute of MIT, MGH, and Harvard, Cambridge, MA USA

**Keywords:** Machine learning, Predictive medicine, MHC class II, Peptide vaccines

## Abstract

CD4^+^ helper T cells contribute important functions to the immune response during pathogen infection and tumor formation by recognizing antigenic peptides presented by class II major histocompatibility complexes (MHC-II). While many computational algorithms for predicting peptide binding to MHC-II proteins have been reported, their performance varies greatly. Here we present a yeast-display-based platform that allows the identification of over an order of magnitude more unique MHC-II binders than comparable approaches. These peptides contain previously identified motifs, but also reveal new motifs that are validated by in vitro binding assays. Training of prediction algorithms with yeast-display library data improves the prediction of peptide-binding affinity and the identification of pathogen-associated and tumor-associated peptides. In summary, our yeast-display-based platform yields high-quality MHC-II-binding peptide datasets that can be used to improve the accuracy of MHC-II binding prediction algorithms, and potentially enhance our understanding of CD4^+^ T cell recognition.

## Abstract (web summary)

Identifying peptides that can bind major histocompatibility complex II (MHC-II) is important for our understanding of T cell immunity and specificity. Here the authors present a yeast-display library screening approach that identifies more potential binders than various reported algorithms to help expand our understanding for antigen presentation.

## Introduction

T cells recognize short, linear peptides displayed by major histocompatibility complexes (MHCs), known as Human Leukocyte Antigens (HLAs) in humans, through their T cell receptors (TCRs). Upon recognition of a cognate peptide-MHC (pMHC) complex, the T cell is activated, initiating an immune response. The resulting immune response can protect against infectious diseases and cancer^1,2^, but this response can also potentiate autoimmunity, allergy, and transplant rejection^3–5^. Proper T cell function also underlies the success of novel antigen-targeted vaccinations and immunotherapies^6–8^.

Given the importance of T cell responses, there is considerable interest in determining which peptides are presented by MHCs for T cell surveillance. The highly polymorphic peptide-binding groove of MHCs and the immense diversity of potential peptide antigens necessitates the use of allele-specific antigen prediction algorithms. Recent advances have described improvements of these computational algorithms^9–11^, their underlying training data^12–14^, or both^15–19^. While these advances have benefited antigen prediction for both class I (MHC-I) and class II MHCs (MHC-II)—canonically recognized by killer CD8^+^ and helper CD4^+^ T cells, respectively—there is sustained interest in improving the performance of MHC-II prediction algorithms^20^, which frequently under-perform their MHC-I counterparts^11,21–25^.

The under-performance of MHC-II prediction algorithms has been at least partially due to a relative paucity of peptide-binding data^26^, as under-performance is particularly pronounced for MHC-II alleles with few reported binders^21,22^. However, peptide binding predictions for even well-characterized MHC-II alleles have under-performed their MHC-I counterparts^21,25^. This is likely due to challenges inherent to class II MHCs, which have more degenerate peptide-binding motifs than their class I counterparts^27^, and an open peptide-binding groove that requires an added algorithmic step of peptide-register determination^22,28–30^. Additionally, publicly available MHC-II-binding peptide datasets contain redundant nested peptide sets and single amino-acid variants of well-characterized peptides, potentially limiting their effective depth and generalizability^26,31^. Therefore, we hypothesize that the under-performance of MHC-II prediction algorithms has been driven by deficiencies in their underlying training data, and can be ameliorated by higher-quality peptide datasets.

Here, we describe a yeast-display-based platform to screen 10^8^ peptides for MHC-II binding, generating over an order of magnitude more unique binders than comparable approaches for two human MHC-II alleles. The identified peptides recapitulate previously reported binding preferences, but also contain additional motifs and important covariances that are not completely captured by other MHC-II peptide datasets. In addition, yeast-display-trained models improve the prediction of peptide-binding affinity for pathogen- and tumor-associated peptides, even when compared to recently described mass spectrometry-based approaches. Collectively, these data show the importance of large datasets of unique peptide binders to improve MHC-II binding prediction, and suggest our approach can potentially facilitate better understanding of CD4^+^ T cell recognition and enhance patient benefit from antigen-targeted therapeutics.

## Results

### Yeast-displayed MHC-II platform identifies peptide binding

**Fig. 1:**
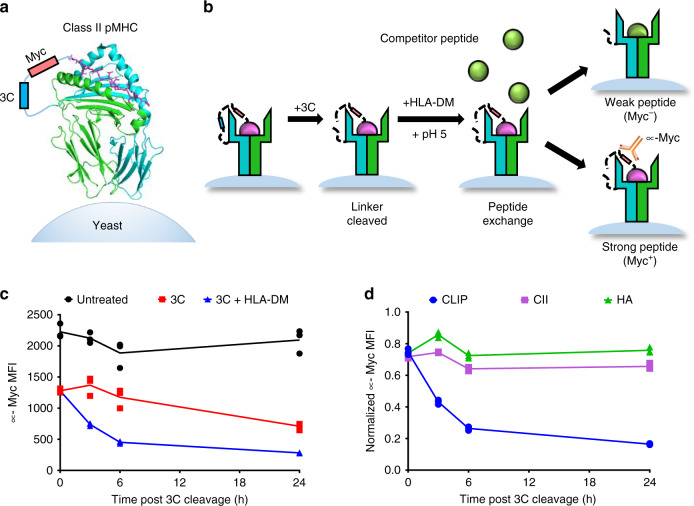
Design and validation of a yeast-display platform to identify peptide binding to a co-expressed class II MHC. **a** Structural representation of HLA-DR401 (PDB 1J8H) modified to encode a 3C protease cleavage site and Myc epitope tag within the linker connecting the peptide and MHC β1 domain. **b** Schematic of validation protocol, including linker cleavage with 3C, peptide exchange at low pH in the presence of HLA-DM and high-affinity competitor peptide, and quantification of remaining bound peptide with an anti-Myc antibody. **c** Time course of mean fluorescence intensity (MFI) of a fluorescently labeled anti-Myc antibody for HLA-DR401-CLIP_81-101_-encoding yeast without treatment (Untreated), with linker cleavage (3C), or with linker cleavage and peptide exchange (3C + HLA-DM), as determined by flow cytometry. **d** Comparison of peptide retention for HLA-DR401-CLIP_81-101_, -CII_261-273_, or -HA_306-318_-encoding yeast with linker cleavage and peptide exchange, as determined by flow cytometry and normalized to MFI before treatment. For each construct, *n* = 3 aliquots were treated independently and measured for each time point and condition. Statistical evaluation was performed by repeated measures two-way ANOVA with Dunnett’s test for multiple comparison within treatment conditions (3 degrees of freedom, F = 54 in 1 C and F = 504 in 1D), or Tukey’s test for multiple comparisons across treatment conditions (2 degrees of freedom, F = 312 in 1 C and F = 2366 in 1D). Source data are provided as a Source Data file.

Yeast-displayed MHC-II constructs have been previously described to probe pMHC-TCR interactions^32,33^. We modified a yeast-displayed HLA-DR401 (HLA-DRA1*01:01, HLA-DRB1*04:01) construct to determine peptide-MHC interactions by introducing a 3C protease site and a Myc epitope tag into the flexible linker that connects the peptide to the HLA β chain (Fig. 1a). Yeast were incubated with 3C protease to cleave the linker, allowing unbound peptides to freely disassociate. Incubation proceeded at low pH in the presence of a high-affinity competitor peptide and the peptide-exchange catalyst HLA-DM (Fig. 1b), emulating the native endosomal environment of MHC-II peptide loading^34^. Yeast encoding binding or non-binding peptides were then differentiated with a fluorescently-labeled antibody directed against the peptide-proximal epitope tag.

Yeast expressing HLA-DR401 linked to the class II-associated invariable chain peptide (CLIP_81-101_), the peptide displaced during endogenous antigen presentation^34^, exhibited significant loss of epitope tag signal immediately following linker cleavage (Fig. 1c). Signal loss increased with incubation at low pH in the presence of a competitor peptide (Fig. 1c). Consistent with its role as a peptide-exchange catalyst, the addition of HLA-DM significantly accelerated signal loss. However, yeast expressing peptides known to more strongly bind to HLA-DR401, HA_306-318_^35,36^ and CII_261-273_^37–39^, retained epitope tag signal when treated with 3C protease and HLA-DM (Fig. 1d), validating our design.

### Selection and analysis of an HLA-DR401 pMHC library

**Fig. 2:**
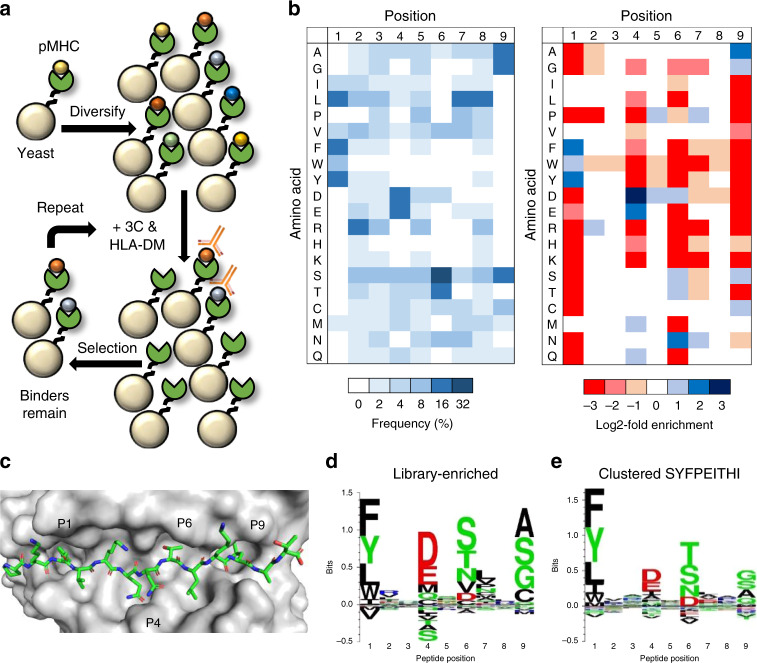
Selection and analysis of a yeast-displayed HLA-DR401 randomized peptide library. **a** Schematic of sequential rounds of library selection to eliminate non-binding peptides and enrich binders. **b** Unweighted heat maps of positional percent frequency and log2-fold enrichment of each amino acid in round 5 of selection (*n* = 81,422 unique peptides). **c** Structure of HA_306-318_ peptide in the HLA-DR401 peptide-binding groove (PDB 1J8H), with primary peptide MHC anchor positions denoted. **d** Kullback–Leibler relative entropy motifs of the core nine amino acids of HLA-DR401-binding peptides, as determined empirically from our yeast-display library, or by clustering of binders curated on the SYFPEITHI database.

To enable repertoire-scale identification of HLA-DR401-binding peptides, we generated a yeast surface display library encoding 1 × 10^8^ random MHC-linked peptides. To simplify downstream analysis, peptides were designed as randomized 9mers flanked by constant residues to favor MHC binding in a single register, as the open MHC-II peptide-binding groove accommodates many possible peptide registers^23,24^. The library was subjected to iterative rounds of linker cleavage, peptide exchange, and selection for epitope tag retention (Fig. 2a), resulting in a pool of yeast encoding strong binders after five rounds (Supplementary Fig. 1A). Upon deep sequencing, we observed rapid convergence upon a peptide motif that was strongly enriched for predicted binders (Supplementary Fig. 1B and 1C). The enriched peptides were highly diverse, consisting of 81,422 unique peptides in the expected register (Supplementary Data 1). The distribution of peptide frequency in the enriched library was largely flat, with no observed correlation between individual peptide frequency and affinity (Supplementary Fig. 2A-C).

We observed strong amino acid preferences at MHC ‘anchor’ peptide positions P1, P4, P6, and P9 (Fig. 2b), where the peptide backbone orients amino acid side chains directly into pockets of the MHC surface (Fig. 2c)^29^. These enrichments largely matched previous reports for HLA-DR401:^17,18,38,40–44^ the deep P1 pocket favors large hydrophobic residues; the basic P4 pocket favors acidic residues; P6 favors polar residues Ser, Thr, and Asn; and the shallow P9 pocket favors Ala, Gly, and Ser. However, the observed enrichment of P9 Cys has not been previously reported, and the enrichment of P6 Asp only aligns with a subset of previous reports^17,18,44,45^. We also observed a less stringent preference for Pro and Asn at P7, which is considered to be an auxiliary anchor position^46^. While the remaining positions are considered to be determinants of TCR binding^47^, each displayed marked preferences, such as the uniform depletion of Trp, the enrichment of Pro and Asp at P5, the strong depletion of P2 Pro, and a previously described preference for P2 Arg^38,41^. Each described enrichment or depletion was highly statistically significant (*p* < 0.001, Supplementary Data 2). Overall, our library-enriched motif (Fig. 2d) closely resembled that of known HLA-DR401 binders (Fig. 2e), generated by clustering previously reported HLA-DR401-binding peptides curated on the SYFPEITHI database^31^.

In order to quantify the impact of the peptide-exchange catalyst HLA-DM on our observed peptide repertoire, we repeated selections in the absence of HLA-DM. With the exception of minor differences in their magnitudes, the observed enrichments and depletions were consistent with HLA-DM addition (Supplementary Fig. 2D, E), suggesting that HLA-DM selects for the retention of high-affinity peptides uniformly across each position, but does not impart unique positional preferences, consistent with previous reports^18,48^.

### Analysis of peptide motifs that deviate from predictions

**Fig. 3:**
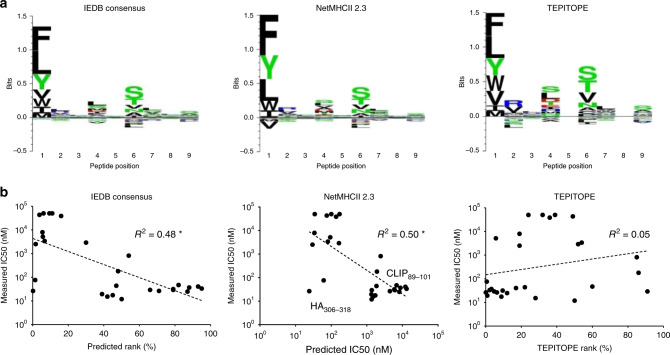
Comparison of library-enriched HLA-DR401-binding motif to MHC-II prediction algorithms. **a** Kullback–Leibler relative entropy motifs of the core nine amino acids of HLA-DR401-binding peptides, as determined by application of selected MHC-II prediction algorithms to computationally-generated peptides. **b** Scatterplots of algorithmic predictions versus measured IC_50_ with lines of best fit and their associated coefficients of determination (*R*^2^). Asterisk denotes *R*^2^ values of negative correlations. Source data are provided as a Source Data file.

While the peptide motifs we observed for HLA-DR401 (Fig. 2d) largely conformed to those observed in previously collected data (Fig. 2e), these motifs did not precisely match those predicted by commonly used MHC-II prediction algorithms, based upon either peptide binding assays, such as NetMHCII 2.3^11^ or IEDB consensus^49^, or upon structural modeling, such as TEPITOPE^50^ (Table 1), especially at P4 and P9 (Fig. 3a).

**Table 1:** Validation of library-enriched HLA-DR401-binding motif. Table of peptides either enriched by our randomized 9mer HLA-DR401 library selections, but not predicted to bind HLA-DR401 by NetMHCII 2.3 or IEDB Consensus, or derived from Influenza A virus and predicted to bind HLA-DR401 but not matching our enriched motif, with algorithmic prediction values and IC_50_ values measured via fluorescence polarization competition assays.

Peptide	Measured IC_50_ (nM)	NetMHCII 2.3 IC_50_ (nM)	IEDB Consensus Rank (%)	TEPITOPE Rank (%)	NeonMHC2 Rank (%)	NetMHCIIpan 4.0 EL Rank (%)
AAANMDTSLPAWEEG	180	1922	48	86	34	25
AAERKMSVLSAWEEG	817	2444	54	85	28	30
AAGVIDPTMLGWEEG	29	1378	66	91	25	24
AALNVERTCHCWEEG	33	13,045	95	2.2	7.5	28
AALREEHTCKCWEEG	37	8801	88	3.2	4.5	29
AALSLERSCKCWEEG	25	8192	87	12	3.6	52
AALVDDPTCRCWEEG	29	6089	79	4.7	6.9	18
AAVADDFSCRGWEEG	47	6836	82	60	27	34
AAWDPDKTVYGWEEG	44	1866	47	22	0.5	0.6
AAWDPERTCRAWEEG	32	5921	79	9.5	0.7	11
AAWERENDMLGWEEG	15	1480	42	28	0.9	1.9
AAWESSTDLVGWEEG	12	1365	50	50	13.7	4.9
AAWHGEGSQIGWEEG	18	1728	45	8.8	0.3	3.2
AAWHNDPACKGWEEG	41	12,112	93	17	1.1	6.0
AAWVPCGDMVSWEEG	26	4439	71	6.3	7.3	13
AAWVVEHSEVGWEEG	19	1345	39	0.9	0.2	0.5
KGYMFESKSMKLRTQ	38,661	138	16	36	40	28
LFEKFFPSSSYRRPV	> 50,000	172	11	32	9.5	15
NQNIITYKNSTWVKD	43,436	75	3.8	49	45	20
SFFYRYGFVANFSME	> 50,000	35	6.1	24	18	31
SRMQFSSFTVNVRGS	3,381	81	6.7	54	14	12
VSSFQDILLRMSKMQ	> 50,000	101	9.4	39	42	50
VVNFVSMEFSLTDPR	7969	34	5.5	19	8	17
YWKQWLSLRNPILVF	2515	30	1.7	19	2.7	15

To determine whether these differences represented bona fide differences in peptide binding, we identified and synthesized peptides that were enriched by our library but deemed non-binders by both NetMHCII 2.3 and the IEDB consensus tool (predicted IC_50_ > 1 µM, consensus rank >10 ^46,49^). We performed fluorescence polarization competition assays using recombinant HLA-DR401 on the selected peptides to determine their IC_50_ values, which are correlated with their MHC-binding affinities^51^. Each tested peptide had an IC_50_ less than 1 µM, and 14/16 bound stronger than HA_306-318_ (76 nM), a well-characterized high-affinity binder^35,36^ (Table 1). Importantly, the binding of the cysteine-containing peptides was specific, as two allele-mismatched cysteine-containing peptides did not exhibit binding (Supplementary Figure 3B). While this observed strong peptide binding was not predicted by legacy algorithms such as NetMHCII 2.3 or IEDB consensus (Fig. 3b), recently described algorithms that use mass spectrometry-derived eluted pMHC ligands as training datasets, such as NeonMHC2^18^ and NetMHCIIpan 4.0 EL^19^, perform markedly better (Table 1), albeit with some remaining discrepancies.

We further identified 8 peptides derived from Influenza A virus [A/Victoria/3/75 (H3N2)] that were predicted as binders by NetMHCII 2.3 or the IEDB consensus tool (IC_50_ < 200 nM, consensus rank <5), but did not match our enriched motif, largely due to departures at P4 and P9 (Table 1). Each had a measured IC_50_ > 2 µM, and 6/8 bound weaker than CLIP_89-101_. These peptides were also largely predicted to be non-binders by NetMHCIIpan 4.0 EL and NeonMHC2 (Table 1). Overall, there was minimal concordance between the measured IC_50_ of these peptides and the predictions of legacy tools such as NetMHCII 2.3, IEDB consensus, or TEPITOPE (Fig. 3b).

These data highlight the importance of high-quality datasets, such as those produced by our yeast-display platform or mass spectrometry, to identify peptides that would have been misclassified by the previous generation of antigen prediction tools.

### Preferences outside of the peptide core affect MHC binding

Canonically, peptide positions P1 through P9 are considered to form the core of the interface with the MHC-II peptide-binding groove^28,29^. However, positions outside of the MHC groove, also known as peptide flanking residues (PFRs), can greatly affect peptide binding^52–54^. Most notably, modifications at position P10 can alter peptide IC_50_ up to two orders of magnitude^53^ without altering the peptide core or TCR interactions^47^.

**Fig. 4:**
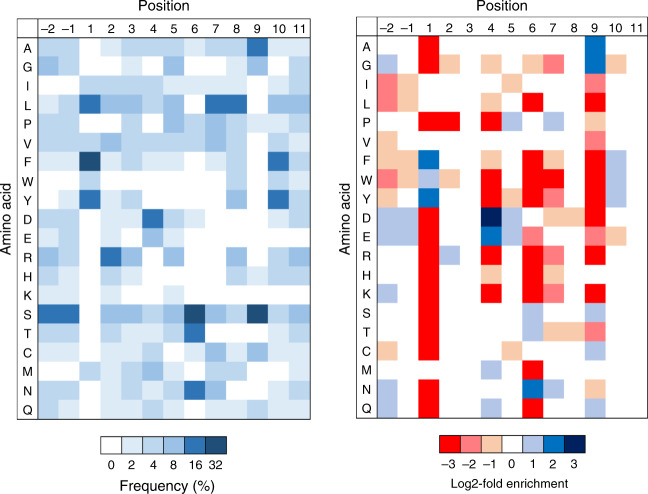
Discovery of preferences at TCR contacts and positions outside the peptide core. Unweighted heat maps of log2-fold enrichment and/or positional percent frequency of each amino acid for all peptides in round five of selection of a randomized 13mer HLA-DR401 library determined to bind in the third peptide register (*n* = 3,374 unique peptides).

To investigate the effect of positions outside of the groove on peptide binding, we constructed and selected a randomized 13mer HLA-DR401 library. While peptides from round 5 displayed no initially obvious motif (Supplementary Fig. 4A), register deconvolution by Gibbs Cluster^55^ identified 7 distinct registers among the 15,147 unique peptides (Supplementary Data 1), 3,374 of which occupied the central register where positions P(-2) through P11 are diversified (Fig. 4). Position P10 displayed a mild preference for aromatic residues, consistent with previous findings^53^, and depletion of both Gly and Glu. We also observed depletion of hydrophobic residues and enrichment of acidic residues at positions P(-2) and P(-1). Positional preferences between positions P1 and P9 were consistent with the original library (Fig. 2b), suggesting our motif was not influenced by the fixed peptide flanking residues in our original design.

To validate these observations, we performed competition assays with variants of CII_261-273_. Notably, modifying P10 to its most enriched residue, tyrosine, resulted in a 30-fold decrease in measured IC_50_, transforming CII_261-273_ into a strong binder (Table 2, Supplementary Fig. 4B). Furthermore, modification to its most depleted residue, glycine, resulted in a 4-fold increase in IC_50_. Added modification of P(-2) and P11, which sit outside the groove but are not considered TCR contacts^47^, did not further benefit peptide binding for favorable residues, but furthered loss of binding for unfavorable residues. We observed comparable effects from modifying each TCR contact [P(-1), P2, P3, P5, and P8] to favorable or unfavorable residues, and the singular modification of P2 to Pro resulted in the loss of any detectable binding, consistent with its strong depletion. Although NetMHCII 2.3 and the recently described NetMHCIIpan 4.0 reportedly consider PFRs^11,19,30^, we did not observe substantial changes in predicted IC_50_ when positions P(-2), P10, or P11 were modified (Table 2).

**Table 2:** Effect of preferences at TCR contacts and positions outside the peptide core. Table of modified CII_261-273_ peptides with associated measured IC_50_ values and the predictions of selected MHC-II prediction algorithms.

Peptide	Positions modified	Measured IC_50_ (nM)	NetMHCII 2.3 IC_50_ (nM)	NetMHCIIpan 4.0 EL Rank (%)
AAGFKGEQGPKGEPG	—	2910	165	0.3
AAGFKGEQGPKGYPG	P10	115	138	0.4
AGGFKGEQGPKGYNG	P(-2), P10, P11	134	100	0.4
AAGFKGEQGPKGGPG	P10	12,101	161	0.4
AIGFKGEQGPKGGVG	P(-2), P10, P11	23,274	144	0.6
AAEFRNEDGPLGEPG	TCR Contacts	80	636	0.3
AAFFGWEWGPDGEPG	TCR Contacts	5,668	4,540	28
AAGFPGEQGPKGEPG	P2	> 50,000	12,348	31

These data demonstrate that peptide binding is greatly affected by positions outside the MHC groove, especially at P10, highlighting additional factors that may be rectified by datasets such as those generated by yeast-displayed libraries.

### Application to a less studied HLA-DR allele

**Fig. 5:**
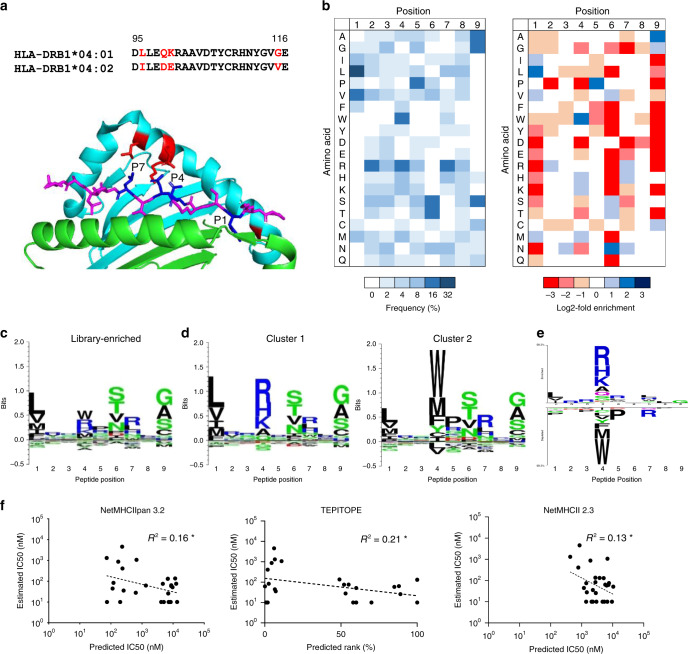
Selection, analysis, and validation of a HLA-DR402 library. **a** Structure of HLA-DR401 complexed with HA_306-308_ (PDB 1J8H) highlighting HLA-DR402 polymorphisms (red) and polymorphism-proximal peptide positions (blue), with associated sequence alignment. **b** Unweighted heat maps of positional percent frequency and log2-fold enrichment of each amino acid in round 5 of selection of a randomized 9mer HLA-DR402 library (*n* = 7,692 unique peptides). **c**, **d** Kullback–Leibler relative entropy motifs of the core nine amino acids of HLA-DR402-binding peptides, either determined empirically from our yeast-display library, or in each of the two clusters found within our library. **e** Amino acids significantly (*p* < 0.05) more represented at each position within the core 9 amino acids of HLA-DR402-binding peptides between clusters. Displayed size of residues correlates with statistical significance of deviation and significance was determined by two-sided unweighted binomial test for *p* < 0.05, with a Bonferroni correction for multiple hypothesis testing. **f** Scatterplots of algorithmic predictions versus measured IC_50_ with lines of best fit and their associated coefficients of determination (*R*^2^). Asterisk denotes *R*^2^ values of negative correlations. Source data are provided as a Source Data file.

Among human MHC-II alleles, HLA-DR401 is well studied, with over 5,000 peptides curated in the Immune Epitope Database (IEDB)^26^. However, many alleles have few, or no, reported binders. We therefore sought to apply our platform to one such allele, HLA-DR402 (HLA-DRA1*01:01, HLA-DRB1*04:02), that differs from HLA-DR401 at four amino acids (Fig. 5a), yet has only 256 peptides curated in the IEDB, many of which are non-unique nested sets and single amino-acid variants of a parental sequence^26,31^.

Our yeast-displayed HLA-DR402 construct was validated through its ability to specifically retain previously reported peptide binders^44,56–60^ (Supplementary Fig. 5A), and a randomized 9mer HLA-DR402 library was constructed, selected, and analyzed. While the predicted affinity of enriched peptides increased throughout selection, the final proportion of predicted binders was low (27%), suggesting a large divergence between our enriched library and prediction algorithms (Supplementary Fig. 5B). Sequences from round 5 of selection again revealed a strongly enriched motif (Fig. 5b), with 7,692 unique peptides in the expected register (Supplementary Data 1).

Consistent with the location and nature of the polymorphisms of HLA-DR402 (Fig. 5a), residue preferences at peptide positions P2, P3, P6, P8, and P9 mirrored those of HLA-DR401, yet differed notably at positions P1, P4, P5, and P7 (Fig. 5b, c). Specifically, the truncated P1 pocket favors smaller hydrophobic residues; P4 favors basic residues and large hydrophobic residues Trp and Met; P5 favors Pro as well as basic residues; and P7 favors basic residues, consistent with the consensus of previous reports^38,44,45,57,61–64^. Further analysis revealed that the enriched sequences represented two unique motifs (Fig. 5d): The first, a conventional HLA-DR motif with strong preferences at MHC anchor positions P1, P4, P6, and P9; the second, an unconventional motif dominated by hydrophobic residues at P4, and significantly (*p* < 0.05) less dependent on hydrophobic residues at P1, but more dependent on P5 Pro (Fig. 5e).

Our enriched motif again differed from those generated by legacy prediction algorithms (Supplementary Figure 5C), that reflect the truncation of the P1 pocket and consistent preferences at P6, yet have increased uncertainty at P9. In addition, the dearth of curated peptide training data for this allele results in an inconclusive motif for NetMHCII 2.3. Our enriched motif was supported by competition assays that validated 16/16 library-enriched peptides (measured IC_50_ < 150 nM) that were not predicted to bind HLA-DR402 by both NetMHCIIpan 3.2 and TEPITOPE (Supplementary Table 1, Supplementary Fig. 5D). These peptides were derived from both clusters within our data, supporting each motif. We further identified 8 peptides derived from Influenza A virus and predicted to be strong binders by both NetMHCIIpan 3.2 and TEPITOPE that did not match our overall enriched motif. Interestingly, only 3/8 were found to be weak or non-binders (IC_50_ > 500 nM), possibly due to averaging two overlapping motifs in our data. Notably, the predictions of legacy algorithms showed no correlation with measured IC_50_ (Fig. 5f). However, many of these deficiencies were rectified by recently described algorithms that use mass spectrometry-derived eluted pMHC ligands as training datasets, such as NeonMHC2 and NetMHCIIpan 4.0 EL (Supplementary Table 1).

These results demonstrate that our platform can generate large quantities of high-quality training data even for alleles for which there are no allele-specific reagents to validate fold and function. It further revealed that HLA-DR alleles can bind peptides in multiple distinct peptide motifs, including non-conventional motifs, and can introduce inaccuracies in algorithms that overweight hydrophobic preferences at position P1.

### Benchmarking performance of yeast-display trained algorithms

We hypothesized that yeast display-derived peptide binding data could be used to improve algorithmic prediction of MHC binding. To address this hypothesis, we trained prediction algorithms with our yeast-displayed library data using NN-Align, the architecture underlying NetMHCII and NetMHCIIpan^64^, facilitating direct comparison of the effect of the training data versus that of the prediction algorithm architecture. Algorithms trained on yeast-display data exhibited good correlation with the above described measured IC_50_ values (Supplementary Fig. 6A, B), and correctly classified 24/24 of the previously measured HLA-DR401 peptides and 21/24 of the HLA-DR402 peptides as binders or non-binders (rank <10% and measured IC_50_ < 1 µM, or rank >10% and measured IC_50_ > 1 µM, respectively). Furthermore, consistent with the effect of peptide flanking residues on binding, training on the 13mer HLA-DR401 yeast-display data resulted in improved correlation with measured IC_50_ for the CII_261-273_ variant peptides, relative to training on the 9mer library, or to NetMHCII 2.3 (Supplementary Fig. 6C).

We next set out to comprehensively benchmark the predictive performance of our algorithm as compared to a large array of other described approaches. We identified two peptide-binding datasets for each allele that were not represented in most current prediction training datasets^18,44^, facilitating independent evaluation. These datasets were generated from eluted ligand mono-allelic mass spectrometry (MS) obtained from antigen-presenting cells that express only a single MHC-II allele, eliminating the ambiguity in allelic assignment encountered in conventional poly-allelic MS eluted ligand datasets^14,27^. This method has recently been used to generate high-quality data for many MHC-I and MHC-II alleles^14,15,18,44^. While these datasets are over an order of magnitude smaller than those generated by yeast-display in terms of unique peptide cores (Supplementary Data 1, Supplementary Fig. 7), their motifs are largely consistent with yeast-display, with the exception of P9 Cys and the absence of two distinct motifs for HLA-DR402. As one of these datasets^18^ underlies the recently published MHC-II prediction algorithm NeonMHC2, we generated an additional prediction algorithm from this data—again using NN-Align—to provide further comparison on the effect of training data versus the underlying algorithmic architecture.

Each algorithm was applied to the remaining allele-matched dataset^44^, with length- and expression-matched decoy peptides, to determine two metrics of predictive performance: the area under the receiver operating characteristic curve (AUC), and the positive predictive value (PPV). While the MS- and 9mer yeast-display-trained models performed comparably to one another, the overall predictive performance of each algorithm was initially relatively low, with a maximum AUC of 0.81 (Supplementary Fig. 8A), suggesting a disparity between the training and evaluation sets. Unsupervised clustering of each MS-derived evaluation set with Gibbs Cluster^55^ revealed that a substantial portion of each set (26% for HLA-DR401, 19% for HLA-DR402) were outliers (Supplementary Data 1), including peptides with long stretches of Gly or Pro, which have been previously reported to nonspecifically populate eluted ligand datasets^65^.

**Fig. 6:**
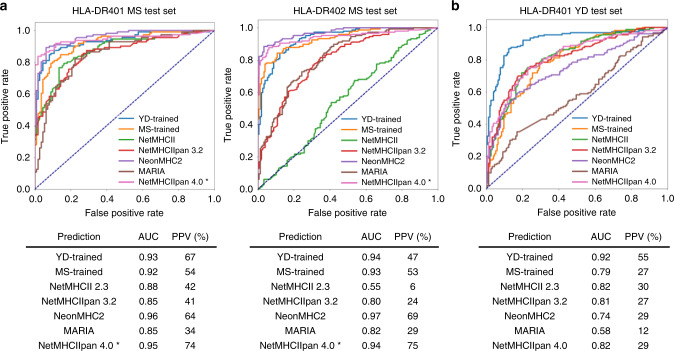
Benchmarking performance of yeast-display-trained algorithms. Receiver operating characteristic (ROC) curves for prediction with existing prediction algorithms, or algorithms trained on our 9mer yeast-display library (YD-trained) or eluted ligand mono-allelic mass spectrometry (MS-trained) data, on either **a** outlier-removed eluted ligand MS data for HLA-DR401 and -DR402, with expression-matched decoy peptides, or **b** yeast-display 13mer HLA-DR401 library data, with naïve library decoys. For each dataset, the area under the ROC curve (AUC) and positive predictive value (PPV) of each prediction are shown. Asterisks indicate algorithms that contain the evaluation set in their training data. Source data are provided as a Source Data file.

Removal of these outliers yielded universally improved prediction performance (Fig. 6a). For both alleles, the MS- and yeast-display-trained algorithms performed comparably in AUC (0.92-0.94), and outperformed NetMHCII 2.3 and NetMHCIIpan 3.2, which are also built on NN-Align. This outperformance was more pronounced in PPV, with the yeast-display-trained algorithm reaching 67% PPV for HLA-DR401. While the recently released NetMHCIIpan 4.0 EL^19^ greatly outperformed its predecessors, its training set included our evaluation set, and therefore this algorithm could not evaluated equitably. NeonMHC2 demonstrated strong performance for both alleles via AUC (0.96–0.97) and PPV (64–69%). As NeonMHC2 is built upon the same underlying data as the MS-trained algorithms, its improved performance may be due to the incorporation of peptide processing information, such as peptide cleavage preferences^18^. In addition, the recently described MixMHC2Pred^14^, which is trained on conventional poly-allelic eluted ligand MS data, displayed comparable performance to NeonMHC2 on a subset of HLA-DR401 peptides (Supplementary Fig. 8B), but could not be fully compared due to peptide length constraints and the absence of an HLA-DR402 predictor. All algorithms evaluated outperformed another recently released poly-allelic eluted ligand MS-trained algorithm, MARIA^16^.

Importantly, however, the use of a MS-derived test set in evaluating predictive performance may not fully capture false negatives that might arise due to gaps in MS-derived data, such as those arising from systemic under-sampling of cysteine-containing peptides^18,44^. Therefore, we further evaluated the predictive performance of each algorithm on our 13mer HLA-DR401 library data. We observed comparable performance between NetMHCII 2.3, NetMHCIIpan 3.2, NetMHCIIpan 4.0 EL, and the MS-trained NN-align algorithm (AUC 0.79-0.82, PPV 27-30%) (Fig. 5b). NeonMHC2 slightly underperformed its NN-Align-based counterpart, even though it was used in ‘tiling mode’ which ignores peptide cleavage preferences, further suggesting that the incorporation of peptide cleavage preferences underlie its previously noted outperformance on MS-derived data. The yeast-display-trained model clearly outperformed each alternative algorithm, with an AUC of 0.92 and a PPV of 55%, and prediction performance only minimally improved by the removal of outlier peptides (Supplementary Figure 8C).

Overall, a yeast-display-trained algorithm performed comparably to current state-of-the-art approaches such as NeonMHC2 and NetMHCIIpan 4.0 on MS-derived data, while performing better on yeast-display-derived data. These results suggest the presence of *bona fide* peptide motifs in yeast-display data that are not adequately sampled in MS-derived data. Direct comparison of the MS- and yeast-display-trained algorithms at a positional level revealed a significantly (*p* < 0.05) more stringent P9 preference in the yeast-display-trained algorithm for both alleles (Supplementary Fig. 7B). Furthermore, consistent with its under-representation in MS-derived data, Cys was significantly over- or under-represented at multiple positions and the MS-trained algorithms had a greater preference for small hydrophobic residues Ile, Leu, and Val at multiple positions.

### Yeast display trained algorithms improve antigen prediction

To investigate the effect these differences may have on the prediction of clinically relevant peptides, we performed antigen prediction for HLA-DR401 with NeonMHC2 and the 9mer yeast-display-trained algorithm on two datasets: the proteome of Influenza A virus (IAV), and expression-validated mutations from human lung adenocarcinoma patients^66^. From these datasets, the 9mer yeast-display-trained model differentially classified—relative to NeonMHC2—5 IAV-derived peptides as strong or non-binders, and differentially classified 13 adenocarcinoma-derived peptides as potential cancer neoantigens. Interestingly, these algorithms displayed non-overlapping algorithmic misses (Supplementary Table 2, Supplementary Figure 9), suggesting that there are peptide motifs unique to both the MS- and yeast-display-derived training data that contribute to improved peptide prediction performance.

**Fig. 7:**
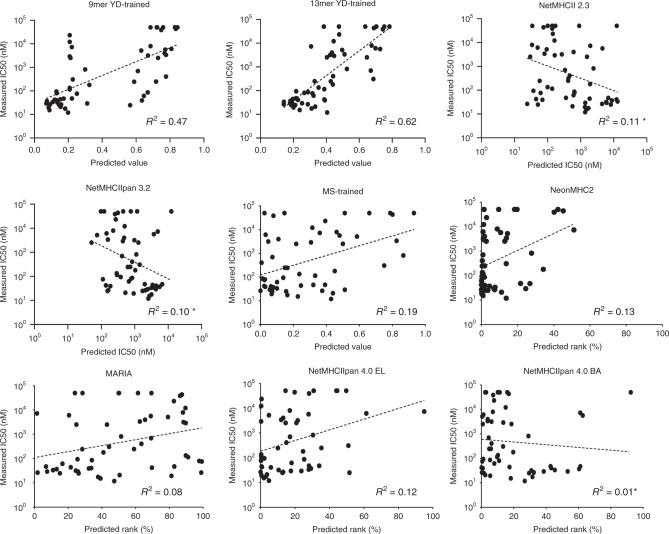
Benchmarking MHC-II algorithm performance for prediction of peptide-binding affinity. Scatterplots of algorithmic predictions versus measured IC_50_ values for 55 peptides assayed for binding to HLA-DR401 in fluorescence polarization competition assays, with lines of best fit and their associated coefficients of determination (*R*^2^). Asterisk denotes *R*^2^ values of negative correlations. Source data are provided as a Source Data file.

When all 55 peptides assayed for binding to HLA-DR401 in this study were considered, current eluted ligand MS-trained algorithms NeonMHC2, NetMHCIIpan4.0 EL, MARIA, and our own MS-trained model displayed little to no correlation with measured IC_50_ (*R*^2^ = 0.08-0.19), indicative of poor peptide affinity prediction performance (Fig. 7). In addition, NetMHCIIpan 4.0 BA, which is trained exclusively on peptide binding affinity data^19^, failed to show correlation with measured IC_50_ for these peptides (*R*^2^ = 0.01) However, our 9mer yeast-display trained model algorithm displayed notably improved correlation with measured IC_50_ (*R*^2^ = 0.47), and consistent with our findings on peptide flanking residues, the predictions of the 13mer yeast-display-trained model displayed even greater correlation (*R*^2^ = 0.62). These findings held true when each prediction was converted to percent rank (Supplementary Figure 10).

Overall, our results demonstrated that both eluted ligand MS- and yeast-display-derived peptide datasets improved the performance of MHC-II prediction algorithms relative to legacy datasets, and both identified unique peptide motifs. However, we find that yeast-display provided much larger datasets than eluted ligand MS, and provided notably improved performance in predicting peptide affinity.

## Discussion

The central role of CD4^+^ T cells across infection, cancer, autoimmunity, and allergy motivates a need to predict which peptide antigens can be presented by MHC-IIs. However, MHC-II prediction algorithms can suffer from consequential gaps and inaccuracies in coverage, especially for less characterized alleles^11,21–25^. Here, we present a platform for large-scale identification of diverse MHC-II-binding peptides. We demonstrated that our platform generates over an order of magnitude more unique data than comparable approaches for two human MHC-II alleles and identifies motifs that are missed by both current data collection techniques and frequently used prediction algorithms. We further trained existing algorithms upon our yeast-display library data and used these algorithms to discover bona fide peptide binders that are not predicted by other prediction algorithms.

Analysis of the training data underlying previously described prediction algorithms revealed multiple sources of underperformance. For both alleles studied, we found large numbers of nested and single amino acid variant peptides within curated training sets. While training algorithms account for redundant information from nested sets^30^, their presence diminishes the functional size of the training set. However, single amino acid variants are considered unique peptides, and can therefore impart biases. Furthermore, a systemic absence of cysteine in training sets resulted in substantial algorithmic false negatives for both alleles. While this is likely due in part to an aversion to working with cysteine-containing peptides, it may also be driven by the difficulties inherent to sampling them in mass-spectrometry (MS)^67^. A systemic underrepresentation of acidic residues in the IEDB has also been reported^18^. In comparison, no systematic absences were observed within our yeast-display data (Supplementary Data 1).

In addition, we found that yeast-displayed libraries uniquely benefit from their large size and engineered composition. By engineering randomized peptide libraries with defined flanking residues, we reduced register uncertainty and increased anchor preference resolution. Meanwhile, the large size of our libraries enabled identification of consequential preferences at non-anchor residues, including those outside the peptide-binding groove. Our libraries also enabled us to identify two distinct motifs for HLA-DR402 that were not adequately captured by curated peptides or eluted ligand MS (Fig. 5d). The coexistence of two unique binding motifs, including one of which defies the conventional notion of a hydrophobic P1 residue-driven HLA-DR motif in favor of hydrophobic residue at P4, is unique relative to recent reports of HLA-DR alleles^14,17,18^. The smaller size of the mono-allelic MS-derived dataset and its under-representation of Trp^18^, which dominated this newly-described motif, may account for its absence.

By using our data to train prediction algorithms and benchmark their performance against existing algorithms, we identified key considerations for MHC-II antigen prediction moving forward. First, our results demonstrate that high-quality training data improves the performance of MHC-II prediction algorithms without alteration of underlying training algorithm architectures, especially for less characterized alleles (Fig. 6a). However, there are important opportunities for algorithmic improvement, such as increased focus on peptide flanking residues. Second, we find that each source of data has non-overlapping strengths and weaknesses for improving prediction performance. Therefore, we believe that an ideal MHC-II prediction algorithm may be trained on both high-quality datasets that reflect native processing^67^, such as eluted ligand MS datasets, as well as large and diverse peptide datasets, such as those generated by our yeast-display platform. Third, we highlight the importance of the choice of validation sets for benchmarking prediction algorithms, as frequently used metrics of prediction performance underestimate false negatives due to gaps in test sets, allowing entire classes of peptides to be missed without impacting performance metrics (Fig. 6a, b). Finally, we find that yeast-display-trained algorithms are superior at predicting peptide affinity, which is a crucial consideration in identifying peptides suitable for antigen-targeted therapeutics^6–8^. The non-binary nature of yeast-display data, which is trained on peptides from five rounds of selection, possibly accounts for this key disparity.

Lastly, as this platform does not require allele-specific reagents, we believe it can generate high-quality repertoire-scale data for many additional MHC-II alleles, even those with few curated binders, greatly increasing its applicability. As such, we believe this technology can greatly benefit the field of MHC-II antigen prediction, and therefore the study and application of CD4^+^ T cell recognition across pathogen infection, cancer, and immune disorders.

## Methods

### Yeast-displayed pMHC design and peptide exchange

Full-length yeast-displayed HLA-DR401 (HLA-DRA1*01:01, HLA-DRB1*04:01) with a cleavable peptide linker was based upon a previously described HLA-DR401 construct optimized for yeast display with the mutations Mα36L, Vα132M, Hβ62N, and Dβ72E to enable proper folding without perturbing either TCR- or peptide-contacting residues^33^. The alpha and beta chain ectodomains were expressed as a single transcript connected by a self-cleaving P2A sequence. The peptide was joined through a flexible linker to N-terminus of MHC β1 domain. This construct was further modified to express a 3C protease site (LEVLFQ/GP) and MYC epitope tag (EQKLISEEDL) within the flexible linker, for a total of 32 amino acids between the peptide and β1 domain. HLA-DR402 (HLA-DRA1*01:01, HLA-DRB1*04:02) was generated by modification of this construct with each native HLA-DRβ polymorphism of HLA-DR402. All yeast-display constructs were produced on the pYAL vector as N-terminal fusions to AGA2. All yeast strains were grown to confluence at 30 °C in pH 5 SDCAA yeast media then subcultured into pH 5 SGCAA media at OD_600_ = 1.0 for 48 h induction at 20 °C^68^.

For peptide retention experiments, the linker between peptide and MHC was cleaved with 1 µM 3C protease in PBS pH 7.4 at a concentration of 2 × 10^8^ yeast/mL for 45 minutes at room temperature. After linker cleavage, yeast expressing the pMHC were washed into pH 5 citric acid saline buffer (20 mM citric acid, 150 mM NaCl) at 1 × 10^8^ yeast/mL with 1 µM HLA-DM and a high-affinity competitor peptide at 4 °C to catalyze peptide exchange. HLA-DR401-expressing yeast were incubated with 1 µM HA_306-318_ (PKYVKQNTLKLAT) and HLA-DR402-expressing yeast were incubated with 5 µM CD48_36-53_ (FDQKIVEWDSRKSKYFES) (Genscript, Piscataway NJ). Peptide dissociation was tracked through an AlexaFluor647-labeled ∝-Myc antibody (Cell Signaling Technologies, Danvers MA) on an Accuri C6 flow cytometer (Becton Dickinson, Franklin Lakes NJ). For each construct, *n* = 3 aliquots were treated independently and measured for each time point and condition. Statistical evaluation of dissociation experiments was performed by repeated measures two-way ANOVA with Dunnett’s test for multiple comparison within treatment conditions, or Tukey’s test for multiple comparisons across treatment conditions, in Prism 8.0 (GraphPad Software Inc, San Diego CA).

### Library design and selection

Randomized peptide yeast libraries were generated by polymerase chain reaction (PCR) of the pMHC construct with primers encoding NNK degenerate codons (Supplementary Methods). To ensure only randomized peptides expressed within the library, the template peptide region encoded multiple stop codons. Randomized 9mer libraries were designed as [AAXXXXXXXXXWEEG…] to constrain peptide register and randomized 13mer libraries were designed as [AXXXXXXXXXXXXXG…]. Randomized pMHC PCR product and linearized pYAL vector backbone were mixed at a 5:1 mass ratio and electroporated into electrically competent RJY100 yeast^69^ to generate libraries of at least 1 × 10^8^ transformants. Libraries were subjected to 3C cleavage and peptide exchange for 16–18 h, as described above, and were selected for peptide-retention via binding of ∝-Myc-AlexaFluor647 antibody and magnetic ∝-AlexaFluor647 magnetic beads (Milltenyi Biotech, Bergisch Gladbach, Germany). Selected yeast were re-cultured, induced, and selected for an additional four rounds, for five total rounds of selection.

### Library deep sequencing and analysis

Libraries were deep sequenced to determine the peptide repertoire at each round of selection. Plasmid DNA was extracted from 5 × 10^7^ yeast from each round of selection with the Zymoprep Yeast Miniprep Kit (Zymo Research, Irvine CA), according to manufacturer’s instructions. Amplicons were generated by PCR with primers designed to capture the peptide encoding region through the polymorphic region that differentiates HLA-DR401 from HLA-DR402 (Supplementary Methods). An additional PCR round was then performed to add P5 and P7 paired-end handles with inline sequencing barcodes unique to each library and round of selection. Amplicons were sequenced on an Illumina MiSeq (Illumina Incorporated, San Diego CA) with the paired-end MiSeq v2 500 bp kit at the MIT BioMicroCenter.

Paired-end reads were assembled via FLASH^70^ and processed with an in-house pipeline that filtered for assembled reads with exact matches to the expected length, polymorphic sequences, and 3C protease cleavage site, then sorted each read based on its inline barcode and extracted the peptide-encoding region. To ensure only high-quality peptides were analyzed, reads were discarded if any peptide-encoding base pair was assigned a Phred33 score less than 20, or did not match the expected codon pattern at NNK sites (*n* = any nucleotide, K = G or T). To account for PCR and read errors from high-prevalence peptides, reads were discarded if their peptide-encoding regions were Hamming distance >1 from any more prevalent sequence, Hamming distance >2 from a sequence 100 times more prevalent, or Hamming distance >3 from a sequence 10,000 times more prevalent within the same round, in line with previously published analysis methods^71^. Unique DNA sequences were translated by Virtual Ribosome^72^ and filtered for peptides not encoding a stop codon.

### Heat map visualization of library peptide preferences

Heat maps were generated from filtered sequences from each round to visually represent positional preferences. For each round, the unweighted prevalence of each amino acid at each position was calculated as a percentage. This positional percent prevalence was compared to its matched value in the unselected library to generate log2-fold enrichment values. The significance of deviations from the positional amino frequencies in the unselected library were evaluated using an unweighted two-sided binomial test using 10,000 peptides to establish each distribution in kpLogo^73^, with a Bonferroni correction for multiple hypothesis testing.

For randomized 9mer libraries, these log2-fold enrichment values were used to generate 20 × 9 position-specific scoring matrices (PSSMs) that were used to identify out-of-register peptides in round 5 of selection. Each 15mer peptide was scored in each of its seven possible 9mer registers by the PSSM, without positional weighting. Peptides which scored highest in a shifted register, regardless of score, were deemed out-of-register. For the randomized 13mer library, peptide register was determined by Gibbs Cluster 2.0^55^, with settings imported from ‘MHC class I ligands of the same length’, a motif of 13 amino acids, no discarding of outlier peptides, and background amino acid frequencies derived from the data. This allowed visualization of each peptide register independently, without collapsing to a common 9mer motif. The number of unique clusters was determined by maximum Kullback-Leibler distance. Results were comparable between both methods of register determination for the 9mer peptide data.

### Analysis of peptide data from external data sources

External MHC-binding peptide data was curated either from the SYFPEITHI database^31^ or from two previously-published eluted ligand mono-allelic mass-spectrometry (MS) datasets^18,44^. Eluted ligand mono-allelic MS peptide data was analyzed as previously recommended^44^, the minimum epitope of nested peptide sets were filtered for those that did not map to immunoglobulin or HLA proteins. Each dataset was clustered by Gibbs Cluster 2.0^55^ with default settings for ‘MHC class II ligands’, excepting the default removal of outlier peptides, and amino acid frequencies ‘from data’, to identify the core 9mer of each peptide. In each case, Kullback-Leibler distance was maximized for one cluster. For identification of outlier peptides, the default removal of outlier peptides was enabled.

### Generation and comparison of peptide motifs

Kullback-Leibler relative entropy motifs were generated with Seq2Logo 2.0^74^. For yeast-display data, the core 9mers of round 5 sequences were input with background amino acid frequencies derived from their average in their matched unselected library. For externally sourced peptide data, unique core 9mers were input with background frequencies from the UNIPROT^75^ average of each amino acid. Motifs for prediction algorithms were generated by application of each prediction to a computationally-generated set of 50,000 unique 15mer peptides with the UNIPROT average frequency of each amino acid. Prediction with each of NetMHCII 2.3^11^, TEPITOPE^50^, NetMHCIIpan 3.2^11^, the IEDB consensus tool^49^ produced a predicted value and core 9mer. Predicted core 9mers of peptides that met published recommendations for binding (NetMHCII and NetMHCIIpan: IC_50_ < 500 nM, TEPITOPE: rank <6, IEDB Consensus: rank <10) were input into Seq2Logo with UNIPROT average background frequencies.

Statistical comparison of peptide motifs was performed with Two Sample Logo^76^. Significance was determined by two-sided unweighted binomial test for *p* < 0.05, with a Bonferroni correction for multiple hypothesis testing.

### Training of peptide prediction algorithms

Allele-specific MHC-II prediction models were generated from yeast-display library data or from external mono-allelic MS data^18,44^ using NN-Align 2.0^64^. For yeast-display library data, the randomized residues of up to 80,000 sequenced peptides were assigned a target value commensurate with the final round of selection in which they were observed, between 0 and 1, with increasing target value for observation in later rounds. As peptides from the pre-selection library were randomly generated, sequences observed in the pre-selection library but not subsequent rounds served as our negative dataset. The 9mer library data was used for training with default settings for ‘MHC class II ligands’, excepting expected peptide length set to 9 amino acids and expected PFR (peptide flanking residue) length set to 0 amino acids. The 13mer library data was used for trained with default settings, excepting expected peptide length set to 13 amino acids.

For the mono-allelic MS data, curated filtered minimum epitopes were assigned a target value of 1. In order to prevent the algorithm from conflating altered amino acid frequencies arising from MS data collection with peptide-binding preferences, each peptide was scrambled to generate negative instances and assigned a target value of 0, in line with previously published recommendations^18^. These algorithms were trained with default ‘MHC class II ligands’ settings.

Reported prediction values are the inverse of model output prediction values (1-value) for ease of comparison to other prediction algorithms. Percentile ranks were established by comparison of prediction values to the distribution of prediction values generated by applying each prediction to 50,000 computationally generated random 15mer peptides (see above).

### Benchmarking and comparison of prediction algorithms

Prediction algorithms were benchmarked against independently generated allele-specific eluted ligand mono-allelic MS or yeast-display library data, with matched decoy peptides. For the MS datasets, the filtered minimum core epitopes (see above) were classified as positive instances, and length- and expression-matched decoy peptides were randomly selected from a pool of computationally generated peptides, as previously described^18^. For each protein observed within the dataset, we tiled across its sequence with peptide lengths randomly selected from the length distribution of the observed peptides, starting at the first amino acid in the protein and allowing an eight amino acid overlap between subsequent proteins. If the length of the last peptide extended beyond the end of the protein, we randomly shifted the starting amino acid such that the starting amino acid of the first peptide and last amino acid of the final peptide were all within the protein. We randomly selected decoy peptides from this set such that the length distribution of decoy peptides matched that of the positive instances, and that there was no 9mer sequence match with the other decoys or positive instances. For the yeast-display dataset, a randomly selected size-matched set of peptides found enriched in round 5 of selection were classified as positive instances, and decoy peptides were randomly selected from peptides only observed in their respective unselected library.

A 1:1 ratio of positive instances and decoy peptides was used to generate receiver operating characteristic (ROC) curves, where area under the ROC curve (AUC) was calculated with scikit-learn version 0.20.3. A 1:19 ratio of positive instances and decoy peptides was used for calculation of positive predictive value (PPV), and calculated as the fraction of true instances observed in the top 5% of predicted value for each algorithm^18^. AUC and PPV values are provided for the 1-log50k(aff) output of NetMHCII 2.3 and NetMHCIIpan 3.2, and was comparable to the performance of the %Rank output. For NetMHCIIpan 4.0, %Rank_EL was provided, and performs comparably to the Score_EL output. MHC-II binding predictions by IEDB Consensus and TEPITOPE Sturniolo on validation peptides were updated on August 9, 2020.

Prediction algorithms were compared at a positional level by Two Sample Logo^76^. For each comparison, the two algorithms were applied to a common set of 50,000 computationally-generated 15mer peptides (see above). The predicted core 9mer of peptides that rank within the 90^th^ percentile or higher of predicted value for only one algorithm were evaluated against the cores of peptides that rank within the 90^th^ percentile or higher of predicted value for both algorithms. Significance was determined by two-sided unweighted binomial test for *p* < 0.05, with a Bonferroni correction for multiple hypothesis testing.

### Recombinant protein production

Recombinant soluble HLA-DM, HLA-DR401, and HLA-DR402 were produced in High Five (Hi5) insect cells (Thermo Fisher) via a baculovirus expression system, as previously described for other MHC-II proteins^32^. Ectodomain sequences of each chain followed by a poly-histidine purification site were cloned into pAcGP67a vectors. For each construct, 2 µg of plasmid DNA was transfected into SF9 insect cells with BestBac 2.0 linearized baculovirus DNA (Expression Systems, Davis CA) using Cellfectin II reagent (Thermo Fisher, Waltham MA). Viruses were propagated to high titer, co-titrated to maximize expression and ensure 1:1 MHC heterodimer formation, then co-transduced into Hi5 cells and grown at 27 °C for 48–72 h. Proteins were purified from the pre-conditioned media supernatant with Ni-NTA resin and size purified via size exclusion chromatography using a S200 increase column on an AKTAPURE FPLC (GE Healthcare, Chicago IL). HLA-DRB1*04:01 and HLA-DRB1*04:02 chains were expressed with CLIP_81-101_ peptide connected by a 3C-protease-cleavable flexible linker to the MHC N-terminus to improve protein yields.

### Peptide competition assays and IC_50_ determination

The IC_50_ of characterized peptides was quantified with a protocol modified from Yin, L. and Stern, L.J. (2014)^51^. Relative binding values were generated at each concentration according to the equation (FP_sample_ – FP_free_)/(FP_no_comp_ – FP_free_), where FP_free_ is the polarization value of the fluorescent peptide before addition of MHC, FP_no_comp_ is the polarization value with added MHC but no competitor peptide, and FP_sample_ is the polarization value with added MHC and competitor peptide. Relative binding curves were generated and fit by Prism 8.0 (GraphPad Software Inc, San Diego CA) to the equation *y* = 1/(1 + [pep]/IC_50_), where [pep] is the concentration of competitor peptide, to determine the IC_50_ of each peptide, its concentration of half-maximal inhibition.

For each 200 µL assay, 100 nM soluble MHC was combined with 25 nM of fluorescently-modified peptide in pH 5 binding buffer and incubated at 37 °C for 72 h in black 96-well flat bottom plates (Greiner Biotech, Kremsmünster, Austria). Modified HA_306-308_ peptide [APRFV{Lys(5,6 FAM)}QNTLRLATG] was used for HLA-DR401 and modified CD48_36-53_ peptide [AQRIVEWDSR{Lys(5,6) FAM)}SRYG] was used for HLA-DR402. *n* = 3 replicates were performed for each unlabeled peptide (Genscript, Piscataway NJ) concentration, ranging in five-fold dilutions from 20 µM to 1.28 nM. Plates were read on a Tecan M1000 (Tecan Group Ltd., Morrisville NC) with 470 nm excitation, 520 nm emission, optimal gain, and a G-factor of 1.10. An important modification of our protocol is the presence of the MHC-linked CLIP peptide that was released by incubation with 3C protease at a 1:100 molar ratio at room temperature for 1 h prior to dilution into plates. Residual cleaved CLIP peptide at 100 nM is not expected to alter peptide binding.

Due to poor soluble expression of HLA-DR402, the assay for HLA-DR402-binding peptides was limited to two concentrations of unlabeled competitor peptide for this allele. However, we found high correlation between two-point estimated IC_50_ values and those obtained from full titration curve fitting for HLA-DR401.

Lines of best fit between predicted and measured affinity for characterized peptide, and associated determinants of determination (*R*^2^), were generated in Prism 8.0 (GraphPad Software Inc, San Diego CA).

### Reporting summary

Further information on research design is available in the Nature Research Reporting Summary linked to this article.

## Supplementary information

Supplementary Information

Reporting Summary

Description of Additional Supplementary Files

Supplementary Data 1

Supplementary Data 2

## Data Availability

All deep sequencing data was deposited on the sequence read archive (SRA) with accession code PRJNA647875. All processed peptide data can be found in Supplementary Data 1. All other data are available upon request. The UNIPROT and SYFPEITHI databases were utilized in this study. Source data are provided with this paper.

## Source data

Source Data

## Code availability

All scripts used for data processing and analysis, as well as all NN-Align model files, are publicly available at [https://github.com/birnbaumlab/Rappazzo-et-al-2020].
