# Supplementary material for: Repertoire-scale determination of class II MHC peptide binding via yeast display improves antigen prediction
Source: Nat Commun. 2020 Sep 4;11:4414. doi: 10.1038/s41467-020-18204-2 (PMC7473865; doi:10.1038/s41467-020-18204-2)
Supplement: Supplementary file 1 — Supplementary Information [file 41467_2020_18204_MOESM1_ESM.pdf]

Supplementary Information

Repertoire-scale determination of class II MHC peptide binding via yeast display improves antigen prediction

Rappazzo, C.G., Huisman, B.D., & Birnbaum, M.E.

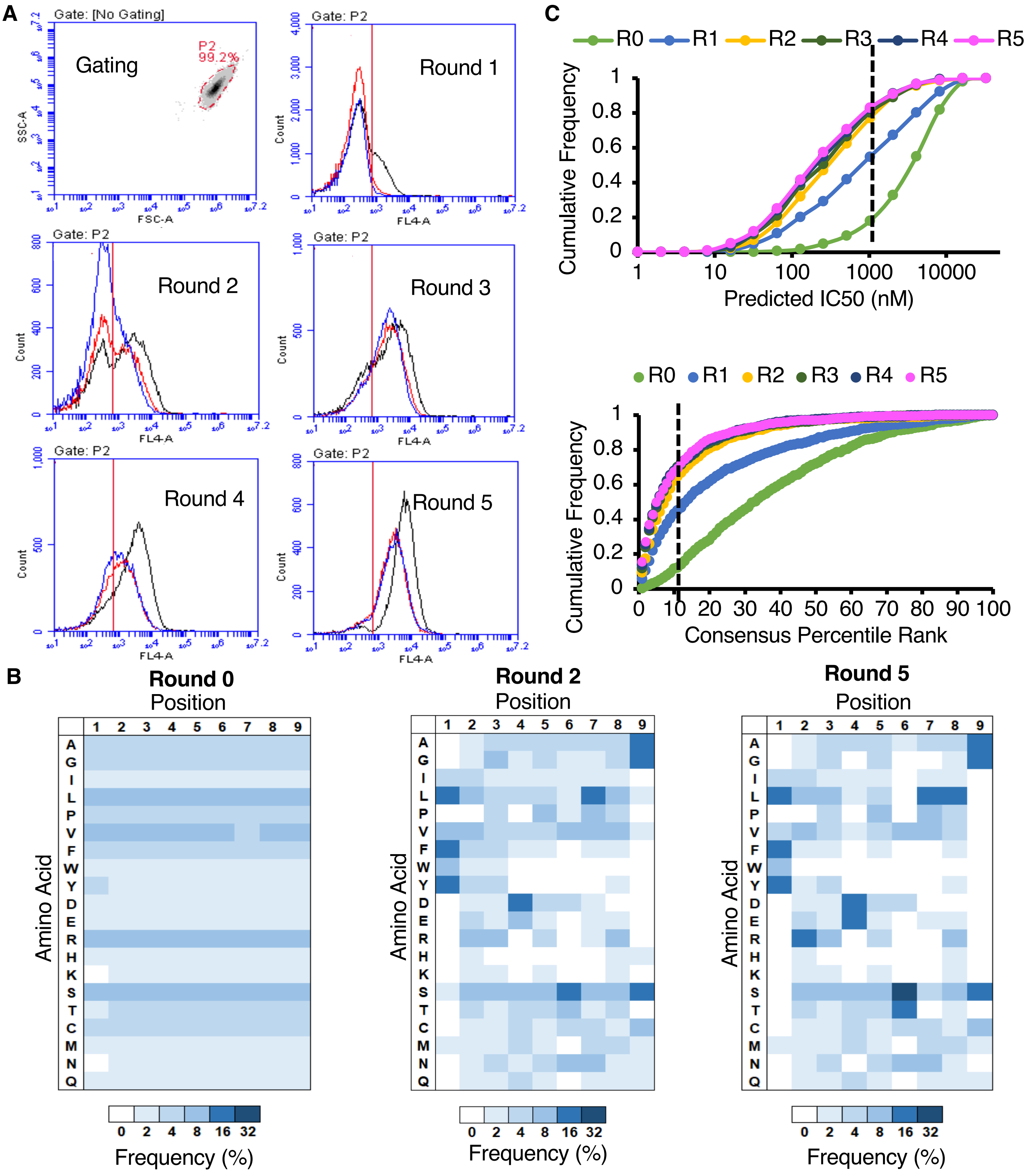

Supplementary Figure 1. Selection of a yeast-displayed HLA-DR401 randomized peptide library. A) Histogram of the fluorescence intensity of a labeled anti-Myc antibody for n = 10,000 yeast in each round of selection either before linker cleavage (Black), following cleavage (Red), or after 24h peptide exchange (Blue), with gating strategy. B) Unweighted heat maps of the positional percent frequency of each amino acid library following round 0 (naïve library), 2, or 5 of selection. C) Cumulative distribution function of predicted peptide IC_{50} (top) and percentile rank (bottom) of 1000 peptides from each round of selection, as determined by NetMHCII 2.3 and the IEDB consensus tool, respectively. Dashed lines represent previously established cut-offs for peptide binding. Source data are provided as a Source Data file.

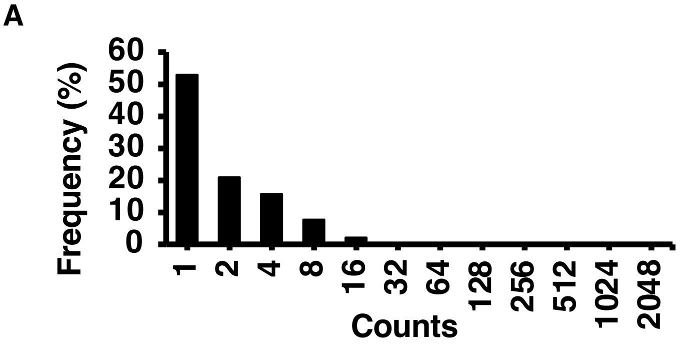

B

Peptide	Counts	IC50 (nM)
AAVNDQLTVNSWEEG	1235	4502
AALGLDLRAYVWEEG	985	7005
AAWVLERGLCCWEEG	648	34
AAFLGEWSLLSWEEG	636	< 10
AARYATSKSKGWEEG	550	9521
AASFLSDSVMGWEEG	534	< 10
AAYSVEPCYRMWEEG	466	37
AAWVRVVPMRAWEEG	414	< 10

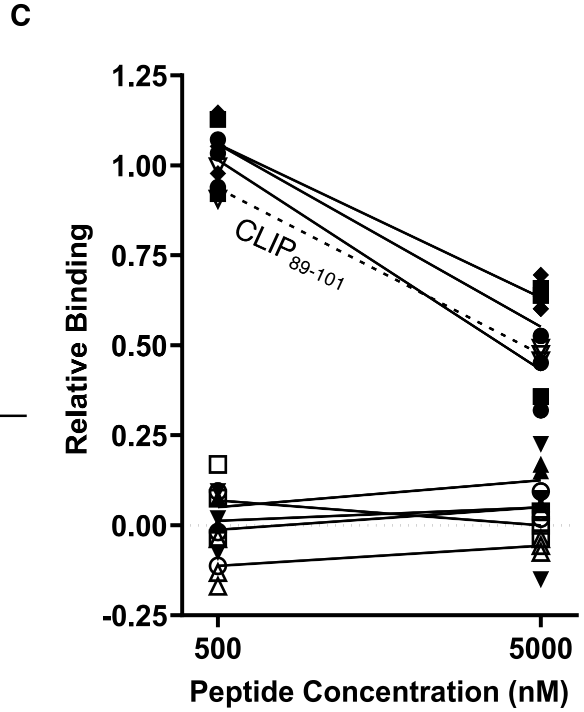

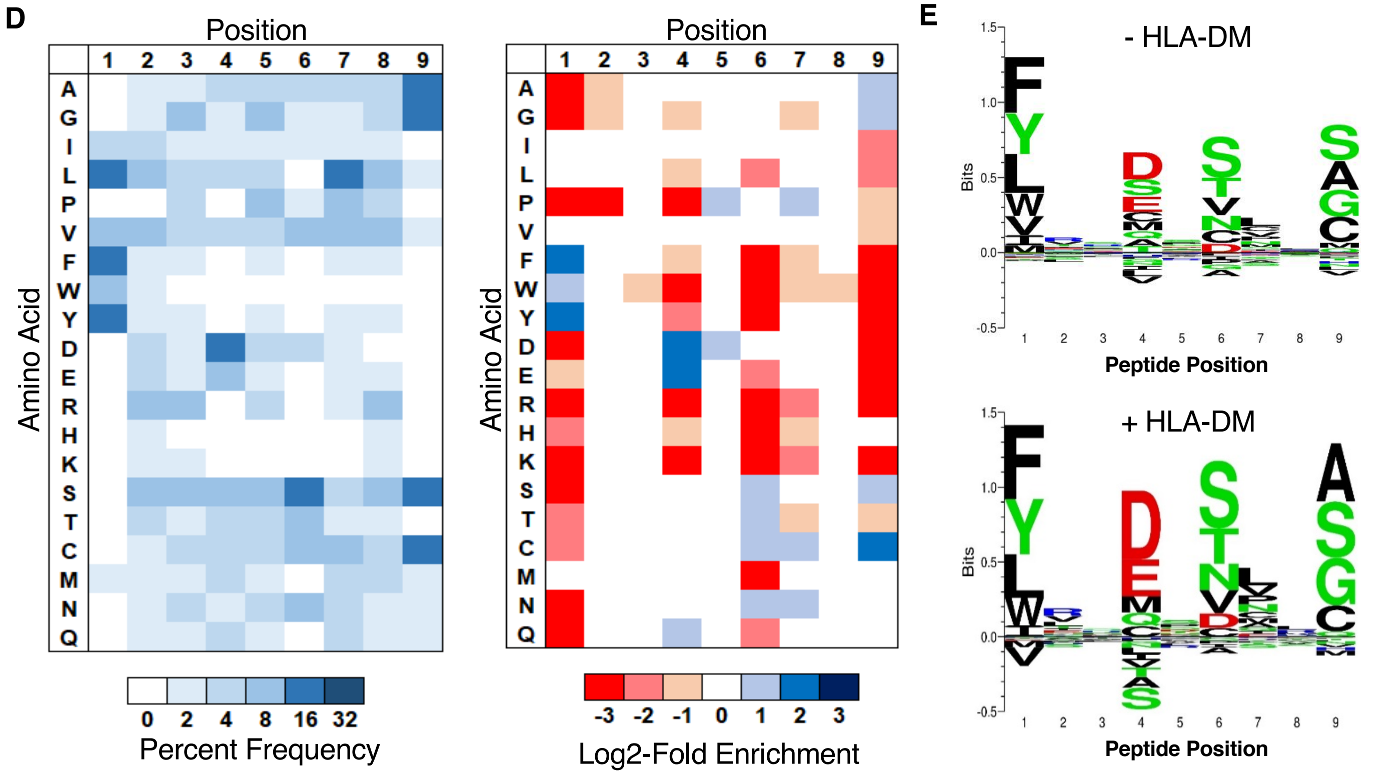

Supplementary Figure 2. Analysis of enriched yeast-displayed HLA-DR401 randomized peptide library. A) Histogram of occurrences of each unique peptide found in round 5 of library selection, with the addition of peptide-exchange catalyst HLA-DM. B) Table of the most enriched peptides found within round 5 of library selection, with occurrences and estimated IC_{50} values derived from two-point fluorescence polarization competition assay binding plots (C). Curves are fit to N = 3 technical replicates per condition. D) Unweighted heat maps of the positional percent frequency and log2-fold enrichment of each amino acid in round 5 of selection without the addition of HLA-DM (N = 105,717 unique peptides). E) Kullback-Leibler relative entropy motifs of the core nine amino acids of HLA-DR401-binding peptides, determined empirically from round 5 of library selection, with or without HLA-DM addition. Source data are provided as a Source Data file.

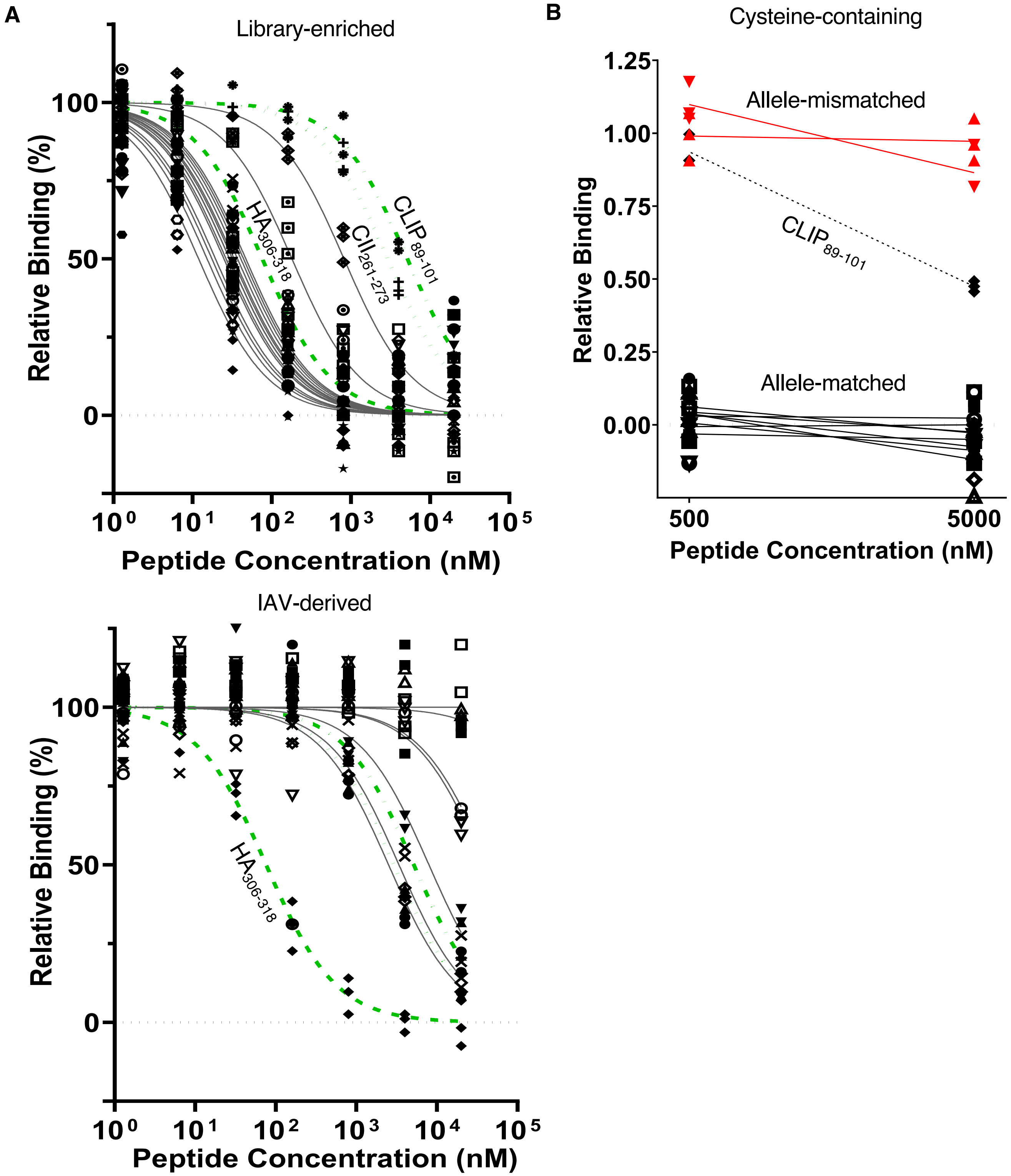

Supplementary Figure 3. Validation of library-enriched HLA-DR401-binding motif. A) Relative binding curves for HLA-DR401 in fluorescence polarization competition assays for peptides either enriched by selection of a 9mer HLA-DR401 library but not predicted to bind HLA-DR401 (Library-enriched) or derived from Influenza A virus and predicted to bind HLA-DR401 but not matching our enriched motif (IAV-derived), with selected control peptides (green). B) Relative binding of cysteine-containing peptides found in round 5 of selection of either the randomized 9mer HLA-DR401 (allele-matched) or HLA-DR402 (allele-mismatched) libraries, tested at two concentrations with HLA-DR401 in a fluorescence polarization competition assay. Curves are fit to N = 3 technical replicates per condition. Source data are provided as a Source Data file.

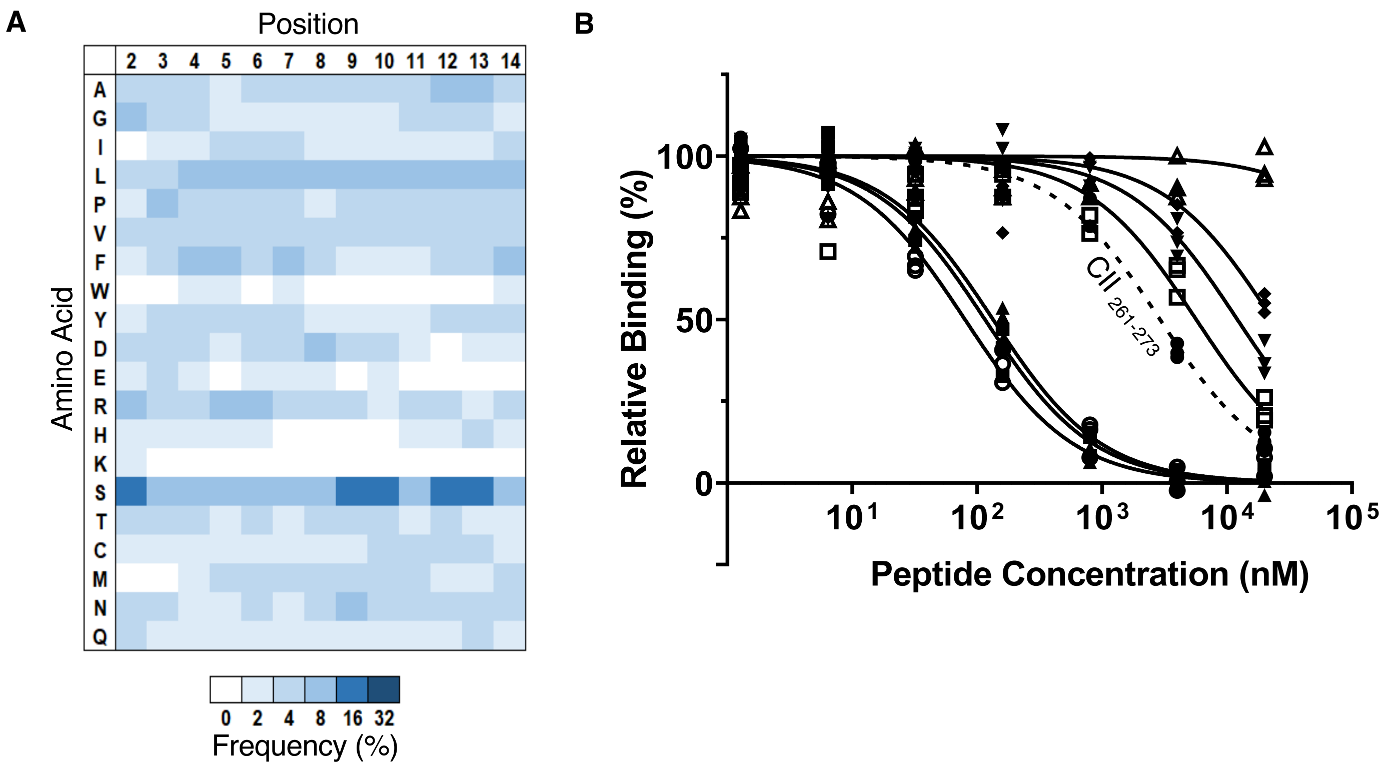

Supplementary Figure 4. Analysis of preferences at TCR contacts and positions outside the peptide core on peptide binding. A) Unweighted heat map of log2-fold enrichment and/or positional percent frequency of each amino acid for all peptides in round five of selection of a randomized 13mer HLA-DR401 library (N = 15,147 unique peptides). B) Relative binding curves for HLA-DR401 fluorescence polarization competition assays of $CII_{261\text{-}273}$ peptide variants. The wild-type peptide is denoted by dashed line. Curves are fit to N = 3 technical replicates. Source data are provided as a Source Data file.

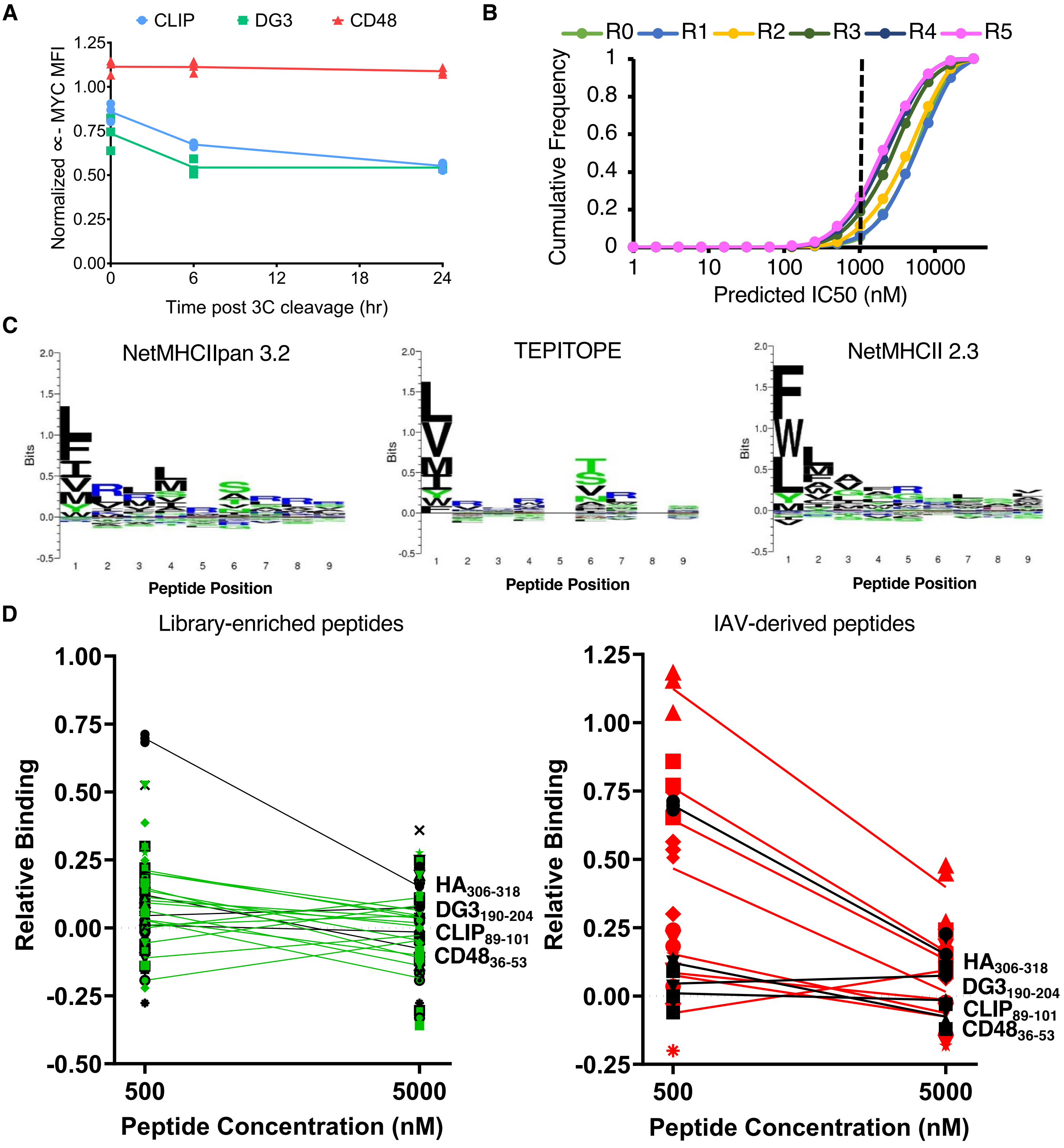

Supplementary Figure 5. Application of yeast-displayed MHC-II platform to a less characterized allele, HLA-DR402. A) Peptide retention for HLA-DR402-encoding yeast, with linker cleavage and peptide exchange, as determined by flow cytometry. B) Cumulative distribution function of predicted IC_{50} of 1,000 peptides from each round of selection of a randomized 9mer HLA-DR402 library, as determined by NetMHCIIpan 3.2. Dashed line represents previously established cut-off for peptide binding. C) Kullback–Leibler relative entropy motifs of the core nine amino acids of predicted HLA-DR402-binding peptides, as determined by application of selected MHC-II prediction algorithms to computationally-generated peptides. D) Two-point fluorescence polarization competition assay binding curves for peptides either found enriched by our randomized 9mer HLA-DR402 library but not predicted to bind HLA-DR402 (Library-enriched) or derived from influenza A virus and predicted to bind HLA-DR402 but not matching our enriched motif (IAV-derived). Selected control peptides are shown in black and curves are fit to N = 3 technical replicates per condition. Source data are provided as a Source Data file.

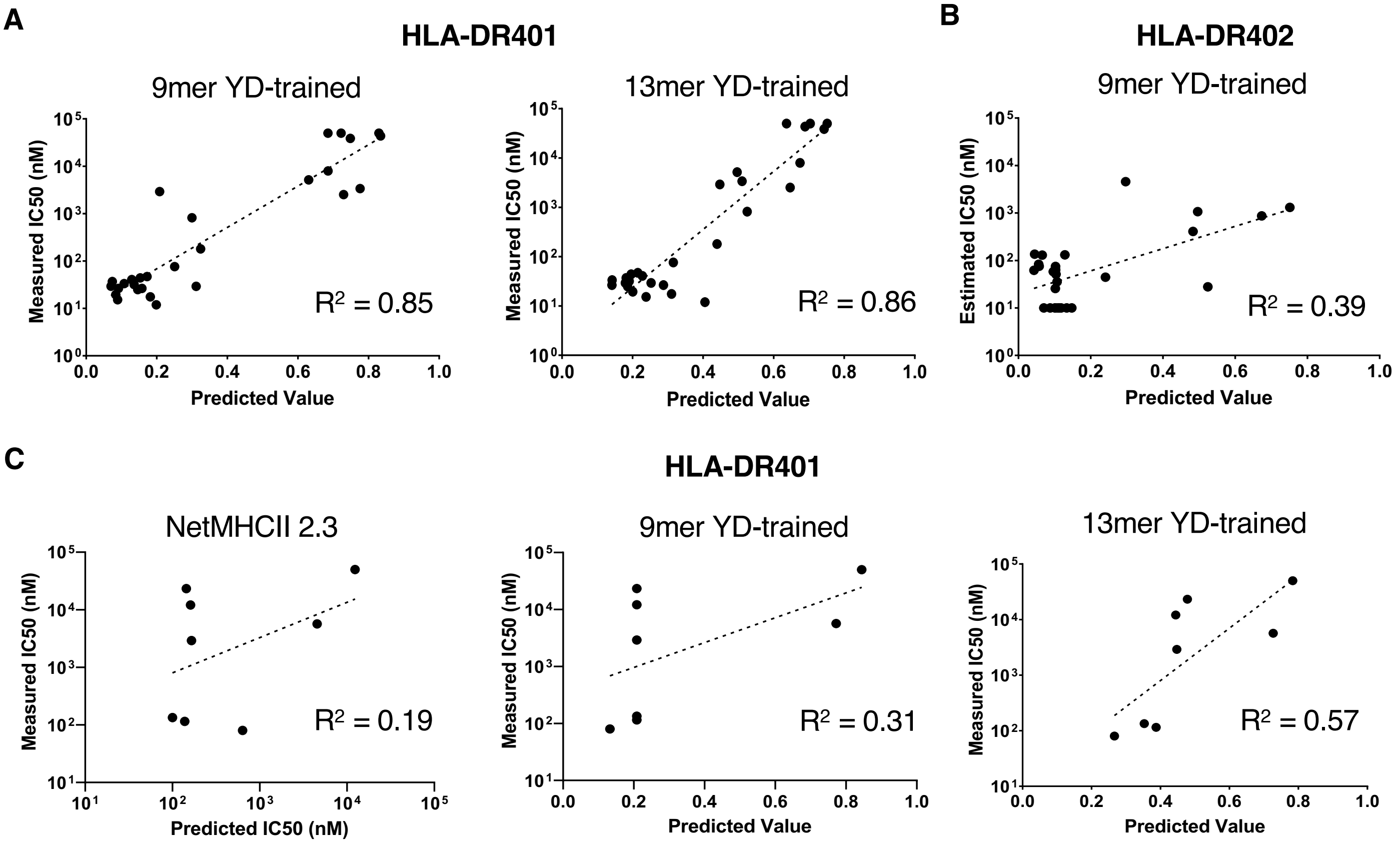

Supplementary Figure 6. Validation of yeast-display library trained MHC-II prediction algorithm. Scatter plots of predicted value and measured IC_{50}, with associated lines of best fit and coefficients of determination (R^2) for the following peptides: (A) Enriched by selection of a 9mer HLA-DR401 library but not predicted to bind HLA-DR401, or derived from Influenza A virus and predicted to bind HLA-DR401 but not matching our enriched motif; (B) Enriched by selection of a 9mer HLA-DR402 library but not predicted to bind HLA-DR402, or derived from Influenza A virus and predicted to bind HLA-DR402 but not matching our enriched motif; C) or variants of wild-type $CII_{261-273}$ peptide. The allele of predicted and measured binding is displayed in bold above each panel. Source data are provided as a Source Data file.

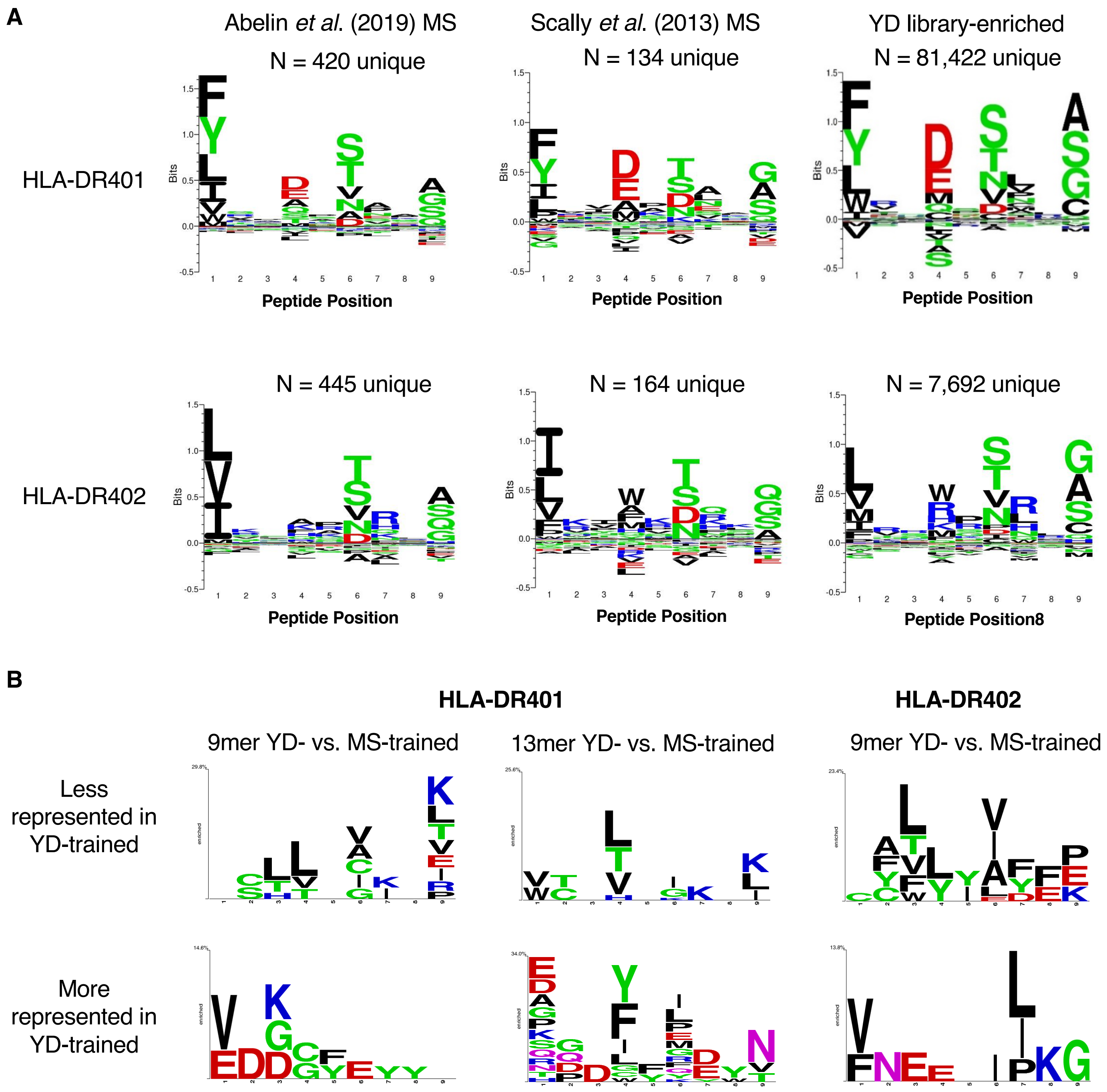

Supplementary Figure 7. Comparison of peptide motifs derived from eluted ligand mono-allelic mass spectrometry and yeast-display library datasets. A) Kullback-Leibler relative entropy motifs of the core 9 amino acids in HLA-DR401 or -DR402-binding peptides, determined from clustering of the filtered minimum core epitopes of nested sets in eluted ligand mono-allelic mass-spectrometry (MS) datasets, or empirically from round 5 of selection of randomized 9mer yeast-display libraries (YD library-enriched). Number of unique cores comprising each motif are shown. B) Amino acids significantly ($p < 0.05$) more or less represented at each position within the core 9 amino acids of HLA-DR401 or -DR402-binding peptides, as determined by algorithms trained on our yeast-display libraries (YD-trained), relative to algorithms trained on eluted ligand mono-allelic MS data (MS-trained). Displayed size of residues correlates with statistical significance of deviation and significance was determined by two-sided unweighted binomial test for $p < 0.05$, with a Bonferroni correction for multiple hypothesis testing

A

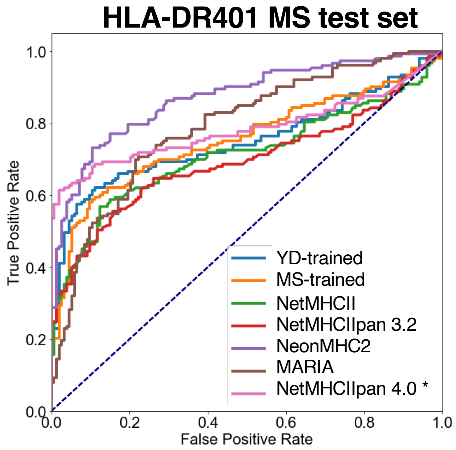

Prediction	AUC	PPV (%)
YD-trained	0.75	50
MS-trained	0.76	45
NetMHCII 2.3	0.71	33
NetMHCIIpan 3.2	0.70	33
NeonMHC2	0.87	49
MARIA	0.79	31
NetMHCIIpan 4.0 *	0.78	58

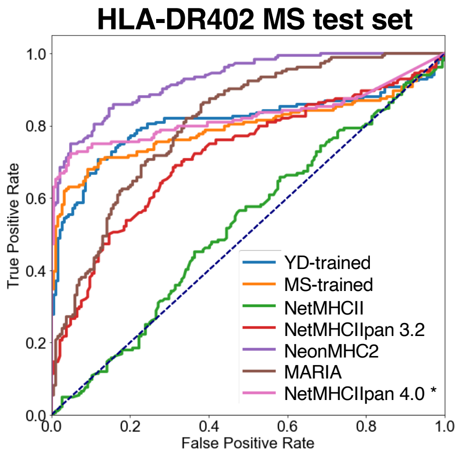

Prediction	AUC	PPV (%)
YD-trained	0.81	43
MS-trained	0.79	45
NetMHCII 2.3	0.53	5
NetMHCIIpan 3.2	0.72	21
NeonMHC2	0.93	59
MARIA	0.81	25
NetMHCIIpan 4.0 *	0.82	64

B

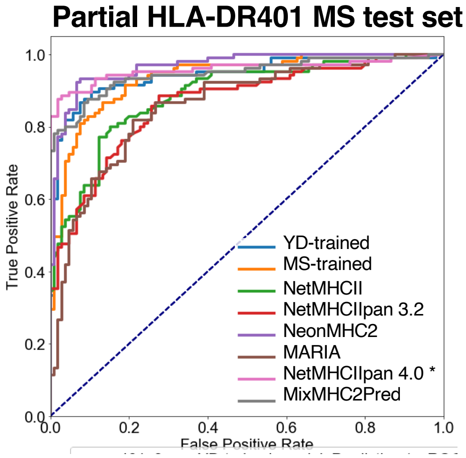

Prediction	AUC	PPV (%)
YD-trained	0.94	70
MS-trained	0.94	61
NetMHCII 2.3	0.89	47
NetMHCIIpan 3.2	0.87	42
NeonMHC2	0.97	68
MARIA	0.86	42
NetMHCIIpan 4.0 *	0.96	77
MixMHC2Pred	0.95	72

C

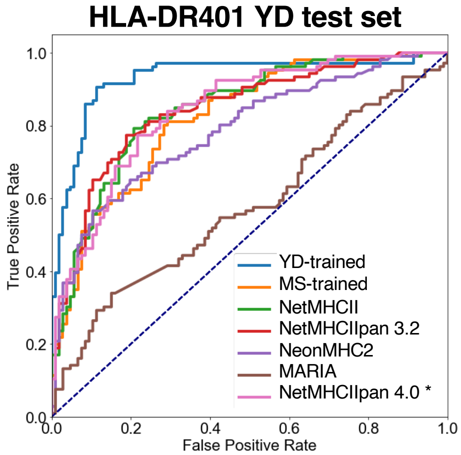

Prediction	AUC	PPV (%)
YD-trained	0.93	58
MS-trained	0.82	29
NetMHCII 2.3	0.84	33
NetMHCIIpan 3.2	0.84	32
NeonMHC2	0.79	32
MARIA	0.58	9
NetMHCIIpan 4.0	0.84	28

Supplementary Figure 8. Benchmarking MHC-II prediction algorithm performance on eluted ligand mass spectrometry or yeast-display data. Receiver operating characteristic (ROC) curves for prediction with published prediction algorithms, or algorithms trained on our 9mer yeast-display library (YD-trained) or mono-allelic MS (MS-trained) data, for either (A) mono-allelic MS data for HLA-DR401 and -DR402, with expression-matched decoy peptides, B) length restricted (12-24mer) outlier-removed mono-allelic MS data for HLA-DR401, with expression-matched decoy peptides or C) outlier-removed round five 13mer yeast-display library data with decoys from the naïve library. For each dataset, the area under the ROC curve (AUC) and positive predictive value (PPV) of each prediction are shown. Asterisks indicate algorithms that contain the evaluation set in their training data. Source data are provided as a Source Data file.

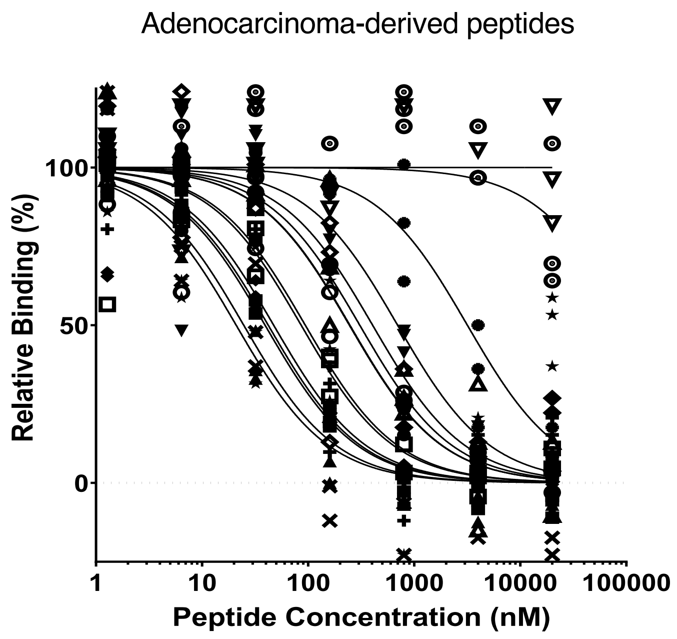

Supplementary Figure 9. Prediction of pathogen- and tumor-associated peptides with MHC-II algorithms trained on yeast-display library or eluted ligand mass spectrometry datasets. Binding curves for HLA-DR401 from fluorescence polarization competition assays for peptides found mutated in human lung adenocarcinomas and differentially predicted as neoantigens for HLA-DR401, or peptides derived from influenza A virus differentially predicted as strong- versus non-binders, by NeonMHC2 or a model trained on our 9mer yeast-display library data. Curves are fit to N = 3 technical replicates per condition. Source data are provided as a Source Data file.

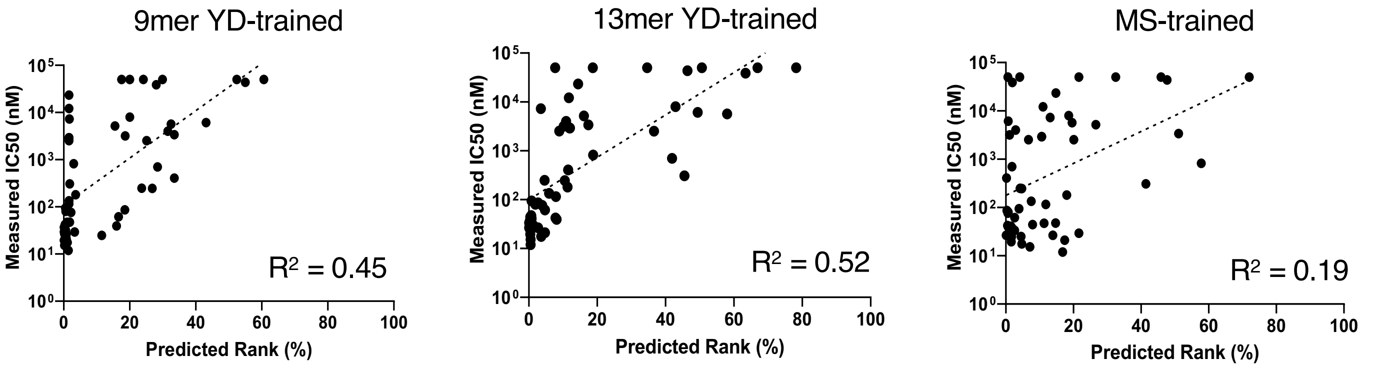

Supplementary Figure 10. Benchmarking MHC-II algorithm performance for prediction of peptide-binding affinity. Scatterplots of algorithmic predictions converted to percentile rank versus measured IC_{50} values for 55 peptides assayed for binding to HLA-DR401 in fluorescence polarization competition assays, with lines of best fit and their associated coefficients of determination (R^2). Asterisk denotes R^2 values of negative correlations. Source data are provided as a Source Data file.

Peptide	NetMHCIIpan 3.2 IC50 (nM)	TEPITOPE Rank (%)	IC50 (nM)	NeonMHC2 Rank (%)	NetMHCIIpan 4.0 EL Rank (%)
AALTGKLGHRGWEEG	5027	70.0	< 10	3.7	29
AAVREKCDHVGWEEG	12551	55.0	76	0.5	14
AAVTNWGCEVGWEEG	11645	49.0	136	9.5	35
AALWRHPGHVGWEEG	4677	52.0	75	3.2	14
AAVSDRLPLRGWEEG	7199	58.0	< 10	14	44
AAVTERPINLGWEEG	9570	60.0	51	18	25
AAVTEEKSHLGWEEG	8693	85.0	58	5.4	5.8
AALDAHRDHMAWEEG	4137	60.0	< 10	0.0	2.4
AAGRSHRTHEGWEEG	13479	100.0	10	2.1	35
AAANRRPSLLGWEEG	8120	100.0	< 10	9.0	75
AAPRRHANHLGWEEG	6738	100.0	130	4.1	21
AATERRPTLMAWEEG	8338	100.0	< 10	16	58
AACRKHGSSIGWEEG	6766	100.0	132	19	89
AAEHVWPSLVGWEEG	6967	89.0	26	8.4	25
AAQVDWPTLPMWEEG	8700	88.0	64	28	41
AAACRKRTWLGWEEG	4992	100.0	< 10	13	80
TMVMELVRMIKRGIN	172	4.5	870	46	41
TEIIRMMESARPEDV	121	2.6	83	9.7	0.6
PALRMKWMMAMKYPI	73	6.6	1310	37	31
YEEFTMVGRRATAIL	229	6.1	4565	30	41
RPMFLYVRTNGTSKI	113	6.6	44	2.8	1.3
LGFVFTLTVPSERGL	235	1.9	409	21	11
NRMVLASTTAKAMEQ	198	0.8	< 10	2.5	1.1
ARQMVQAMRTIGTHP	256	7.2	36	2.5	1.3

Supplementary Table 1. Peptides either found enriched by our randomized 9mer HLA-DR402 library but not predicted to bind HLA-DR402 (top) or derived from influenza A virus and predicted to bind HLA-DR402 but not matching our enriched motif (bottom). Each peptide is displayed with its measured IC50 from two-point fluorescence polarization competition assays, as well as selected algorithmic predictions.

Peptide	WT NeonMHC2 Rank (%)	WT YD-trained Rank (%)	Mut. NeonMHC2 Rank (%)	Mut. YD-trained Rank (%)	Measured IC50 (nM)
LLPVKQSVVPLAKGTLITHC	7.7	5.4	14	1.8	307
ASDSSYRNECPMAEKEDTQM	30	22	22	1.8	47
MKRRPVWTDINPKAVTNDEL	14	4.3	11	0.8	21
PIPVIFHRIATELRKTNDIN	0.8	28	< 0.1	0.4	698
VYGWATLVSERSKNGMQRIL	0.4	27	0.3	0.5	245
YVGDMLAWLHQSTASEKEHL	1.0	46	0.5	34	406
EVKYCTFSKDRSKPIPGMTL	1.3	32	1.5	18	> 50,000
WRAPSYILSPELTQRLFSAA	2.1	39	1.8	24	248
RKDLIVMLMDTDVNKQDKQK	1.8	30	1.0	19	3173
YCDLPQLFRLSCSSTQLNEL	2.1	31	2.0	16	39
FVGNIAEDLCLDITKLSARG	2.4	43	1.6	19	86
ESSISDKNYWKTVSNAFSVI	5.8	25	2.0	12	25
YSIEVLLVLDDSVVRFHGKE	1.4	30	1.2	30	> 50,000
FYHKCDNECMESVRNGTYDY	51	1.7	-	-	7273
LFQNWGVEPIDNVMGMIGIL	13	1.6	-	-	2524
EGIPLYDAIKCMRTFFGWKE	0.1	17	-	-	61
ARQMVQAMRTIGTHPSSSAG	1.7	32	-	-	3994
MFLYVRTNGTSKIKMKWGME	0.9	43	-	-	6102

Supplementary Table 2. Prediction of pathogen- and tumor-associated peptides with MHC-II algorithms trained on yeast-display library or eluted ligand mass spectrometry datasets. Mutant peptides from human lung adenocarcinomas differentially predicted as neoantigens for HLA-DR401, or peptides derived from Influenza A virus differentially predicted as strong- versus non-binders, by NeonMHC2 or a model trained on our 9mer yeast-display library data, with measured IC_{50} values for peptides that NeonMHC2 and the yeast-display trained algorithm disagreed, derived from fluorescence polarization competition assays.

Supplementary Methods

Primer Sequences – Library creation

DR401 13NLibrary For
TATTGCTAGCGTTTTGGCAGCTNNKNNKNNKNNKNNKNNKNNKNNKNNKNNKNNKNNKNNKGGTGGCTCAGGTGGCGG
DR4 NNK1RegLibrary For
TATTGCTAGCGTTTTGGCAGCTGCCNNKNNKNNKNNKNNKNNKNNKNNKNNKTGGGAAGAAGGTGGCTCAGGTGGCGG
DR4 LibExt For Long
CCCTGGACCTATGCAGTTGTTGAGATGTTTTTCTATTTTCTCTGTTATTGCTAGCGTTTTGGCAGCT
pYAL EP Rev
ACCGCCACCACCAGATCCACCACCACCTTTATCGTCATCATCTTTATAATCGGATCC

Primer Sequences – Deep Sequencing

DR401 NNK1Reg Lib R0 Seq For
ACACGACGCTCTTCCGATCTNNNNNNNNATCACGTTCTCTGTTATTGCTAGCGTTTTGGCAG
DR401 NNK1Reg Lib R1 3CDM Seq For
ACACGACGCTCTTCCGATCTNNNNNNNNCGATGTTTCTCTGTTATTGCTAGCGTTTTGGCAG
DR401 NNK1Reg Lib R2 3CDM Seq For
ACACGACGCTCTTCCGATCTNNNNNNNNTTAGGCTTCTCTGTTATTGCTAGCGTTTTGGCAG
DR401 NNK1Reg Lib R3 3CDM Seq For
ACACGACGCTCTTCCGATCTNNNNNNNNTGACCATTCTCTGTTATTGCTAGCGTTTTGGCAG
DR401 NNK1Reg Lib R4 3CDM Seq For
ACACGACGCTCTTCCGATCTNNNNNNNNACAGTGTTCTCTGTTATTGCTAGCGTTTTGGCAG
DR401 NNK1Reg Lib R5 3CDMSeq For
ACACGACGCTCTTCCGATCTNNNNNNNNGCCAATTTCTCTGTTATTGCTAGCGTTTTGGCAG
DR401 NNK1Reg Lib R1 3C Seq For
ACACGACGCTCTTCCGATCTNNNNNNNNCAGATCTTCTCTGTTATTGCTAGCGTTTTGGCAG
DR401 NNK1Reg Lib R2 3C Seq For
ACACGACGCTCTTCCGATCTNNNNNNNNACTTGATTCTCTGTTATTGCTAGCGTTTTGGCAG
DR401 NNK1Reg Lib R3 3C Seq For
ACACGACGCTCTTCCGATCTNNNNNNNNGATCAGTTCTCTGTTATTGCTAGCGTTTTGGCAG
DR401 NNK1Reg Lib R4 3C Seq For
ACACGACGCTCTTCCGATCTNNNNNNNNTAGCTTTTCTCTGTTATTGCTAGCGTTTTGGCAG
DR401 NNK1Reg Lib R5 3C Seq For
ACACGACGCTCTTCCGATCTNNNNNNNNGGCTACTTCTCTGTTATTGCTAGCGTTTTGGCAG
DR401 NNK1Reg Lib Seq Long Rev
GCTGAACCGCTCTTCCGATCTNNNNNNNNTTTAACCTGCTCCAAGAAACGTGGT

DR401 13N Lib R0 Seq For
ACACGACGCTCTTCCGATCTNNNNNNNNAGACACTTCTCTGTTATTGCTAGCGTTTTGGCAG
DR401 13N Lib R1 Seq For
ACACGACGCTCTTCCGATCTNNNNNNNNATGATATTCTCTGTTATTGCTAGCGTTTTGGCAG
DR401 13N Lib R2 Seq For
ACACGACGCTCTTCCGATCTNNNNNNNNCGTGATTTCTCTGTTATTGCTAGCGTTTTGGCAG
DR401 13N Lib R3 Seq For
ACACGACGCTCTTCCGATCTNNNNNNNNTACGTATTCTCTGTTATTGCTAGCGTTTTGGCAG
DR401 13N Lib R4 Seq For
ACACGACGCTCTTCCGATCTNNNNNNNNTGAGCGTTCTCTGTTATTGCTAGCGTTTTGGCAG
DR401 13N Lib R5 Seq For
ACACGACGCTCTTCCGATCTNNNNNNNNTACAGTTTCTCTGTTATTGCTAGCGTTTTGGCAG
DR4 Lib Seq Long Rev
GCTGAACCGCTCTTCCGATCTNNNNNNNNCCGCGGCCCGCT

DR402 NNK1Reg Lib R0 Seq For

ACACGACGCTCTTCCGATCTNNNNNNNNGTGTCATTCTCTGTTATTGCTAGCGTTTTGGCAG

DR402 NNK1Reg Lib R1 Seq For

ACACGACGCTCTTCCGATCTNNNNNNNNTCTGACTTCTCTGTTATTGCTAGCGTTTTGGCAG

DR402 NNK1Reg Lib R2 Seq For

ACACGACGCTCTTCCGATCTNNNNNNNNTGACTATTCTCTGTTATTGCTAGCGTTTTGGCAG

DR402 NNK1Reg Lib R3 Seq For

ACACGACGCTCTTCCGATCTNNNNNNNNAGATGATTCTCTGTTATTGCTAGCGTTTTGGCAG

DR402 NNK1Reg Lib R4 Seq For

ACACGACGCTCTTCCGATCTNNNNNNNNACTCATTTCTCTGTTATTGCTAGCGTTTTGGCAG

DR402 NNK1Reg Lib R5 Seq For

ACACGACGCTCTTCCGATCTNNNNNNNNTCGCTCTTCTCTGTTATTGCTAGCGTTTTGGCAG

DR4 Lib Seq Long Rev

GCTGAACCGCTCTTCCGATCTNNNNNNNNCCGCGGCCCGCT

PE For

AATGATACGGCGACCACCGAGATCTACACTCTTTCCCTACACGACGCTCTTCCGATCT

PE Rev

CAAGCAGAAGACGGCATACGAGATCGGTCTCGGCATTCCTGCTGAACCGCTCTTCCGATCT
