# Supplementary material for: Repertoire-scale determination of class II MHC peptide binding via yeast display improves antigen prediction
Source: Nat Commun. 2020 Sep 4;11:4414. doi: 10.1038/s41467-020-18204-2 (PMC7473865; doi:10.1038/s41467-020-18204-2)
Supplement: Supplementary file 2 — Reporting Summary [file 41467_2020_18204_MOESM2_ESM.pdf]

nature research

Corresponding author(s): Michael Birnbaum

Last updated by author(s): Aug 10, 2020

Reporting Summary

Nature Research wishes to improve the reproducibility of the work that we publish. This form provides structure for consistency and transparency in reporting. For further information on Nature Research policies, see Authors & Referees and the Editorial Policy Checklist .

Statistics

For all statistical analyses, confirm that the following items are present in the figure legend, table legend, main text, or Methods section.

n/a	Confirmed	
☐	☒	The exact sample size (n) for each experimental group/condition, given as a discrete number and unit of measurement
☐	☒	A statement on whether measurements were taken from distinct samples or whether the same sample was measured repeatedly
☐	☒	The statistical test(s) used AND whether they are one- or two-sided<br>*Only common tests should be described solely by name; describe more complex techniques in the Methods section.*
☒	☐	A description of all covariates tested
☐	☒	A description of any assumptions or corrections, such as tests of normality and adjustment for multiple comparisons
☒	☐	A full description of the statistical parameters including central tendency (e.g. means) or other basic estimates (e.g. regression coefficient) AND variation (e.g. standard deviation) or associated estimates of uncertainty (e.g. confidence intervals)
☐	☒	For null hypothesis testing, the test statistic (e.g. F, t, r) with confidence intervals, effect sizes, degrees of freedom and P value noted<br>*Give P values as exact values whenever suitable.*
☒	☐	For Bayesian analysis, information on the choice of priors and Markov chain Monte Carlo settings
☒	☐	For hierarchical and complex designs, identification of the appropriate level for tests and full reporting of outcomes
☒	☐	Estimates of effect sizes (e.g. Cohen's d, Pearson's r), indicating how they were calculated

Our web collection on statistics for biologists contains articles on many of the points above.

Software and code

Policy information about availability of computer code

Data collection	No software was used in the collection of data

Data analysis

Our custom scripts are available on GitHub (https://github.com/birnbaumlab/Rappazzo-et-al-2020; link also provided in Data Availability section). Below are links and versions for external tools and software used in this study:
FLASH (No version) https://ccb.jhu.edu/software/FLASH/
Virtual Ribosome (Version 2.0) http://www.cbs.dtu.dk/services/VirtualRibosome/
kpLogo (No version) http://kplogo.wi.mit.edu/
Seq2Logo 2.0 (Version 2.0) https://services.healthtech.dtu.dk/service.php?Seq2Logo-2.0
Two Sample Logo (No version) http://www.twosamplelogo.org/cgi-bin/tsl/tsl.cgi
SYFPEITHI (Version 1.0) http://www.syfpeithi.de/
TEPITOPE (Sturniolo) http://tools.iedb.org/mhcii/
IEDB Consensus (2.22) http://tools.iedb.org/mhcii/
NNAlign (Version 2.0) https://services.healthtech.dtu.dk/service.php?NNAlign-2.0
NetMHCII (Version 2.3) https://services.healthtech.dtu.dk/service.php?NetMHCII-2.3
NetMHCIIPan (Version 3.2) https://services.healthtech.dtu.dk/service.php?NetMHCIIpan-3.2
NetMHCIIPan (Version 4.0) https://services.healthtech.dtu.dk/service.php?NetMHCIIpan-4.0
MARIA (No version) https://maria.stanford.edu/
MixMHC2Pred (No version) http://mixmhc2pred.gfellerlab.org/
NeonMHC2 (No version) https://neonmhc2.org/neonmhc2/neonmhc2_main/
PRISM (Version 8.0)
IEDB (No version) https://www.iedb.org/
UNIPROT (Release 50.0) https://www.uniprot.org/statistics/UniProt%208
scikit-learn (Version 0.20.3) https://scikit-learn.org/stable/index.html

For manuscripts utilizing custom algorithms or software that are central to the research but not yet described in published literature, software must be made available to editors/reviewers. We strongly encourage code deposition in a community repository (e.g. GitHub). See the Nature Research guidelines for submitting code & software for further information.

Data

Policy information about availability of data

All manuscripts must include a data availability statement. This statement should provide the following information, where applicable:

- Accession codes, unique identifiers, or web links for publicly available datasets
- A list of figures that have associated raw data
- A description of any restrictions on data availability

All deep sequencing data are deposited on the sequence read archive (SRA), with accession code PRJNA647875 (also provided in Data Availability section). All peptide data can be found in Supplemental Data 1. Source data are provided in the Source Data file. All other data are available upon request.

Field-specific reporting

Please select the one below that is the best fit for your research. If you are not sure, read the appropriate sections before making your selection.

☒ Life sciences ☐ Behavioural & social sciences ☐ Ecological, evolutionary & environmental sciences

For a reference copy of the document with all sections, see nature.com/documents/nr-reporting-summary-flat.pdf

Life sciences study design

All studies must disclose on these points even when the disclosure is negative.

Sample size

For yeast peptide retention experiments we chose N=3 replicates for each peptide and treatment condition based on the expected size of differences, and these replicates reveal no differences in retention dynamics. For peptide IC50 determination, N=3 replicates were chosen based on previous studies (Lin, Y. and Stern, L.J., 2014) and no conclusions were drawn in the manuscript based upon differences that could not be captured within the uncertainty of these measurements.

Data exclusions

In next generation sequencing analysis, sequences that contained a stop codon, did not contain an intact protease site, or sequences that were were Hamming distance > 1 from any more prevalent sequence, Hamming distance > 2 from a sequence 100 times more prevalent, or Hamming distance > 3 from a sequence 10,000 times more prevalent were excluded from analyses, as described in the methods section. No other data were excluded from analysis.

Replication

For yeast peptide retention experiments we replicated original findings for N=1 aliquot time course experiments with N=3 aliquots and found no differences in the retention dynamics. For peptide IC50 determination, experiments were first performed in triplicate at two concentrations of unlabeled competitor peptide followed by a full seven concentration titration, also in triplicate. Results between these experiments were highly correlated. Yeast libraries were not replicated because of their time and resource intensiveness of experiments, but results were consistent between the randomized 9mer and 13mer HLA-DR401 libraries, supporting the repeatability of these data.

Randomization

Yeast studies were conducted at population scale so no randomization were warranted.

Blinding

Blinding was not conducted because no qualitative determinations between samples were needed and all experiments were conducted at population scale.

Reporting for specific materials, systems and methods

We require information from authors about some types of materials, experimental systems and methods used in many studies. Here, indicate whether each material, system or method listed is relevant to your study. If you are not sure if a list item applies to your research, read the appropriate section before selecting a response.

Materials & experimental systems

n/a	Involved in the study
☐	☒ Antibodies
☐	☒ Eukaryotic cell lines
☒	☐ Palaeontology
☒	☐ Animals and other organisms
☒	☐ Human research participants
☒	☐ Clinical data

Methods

n/a	Involved in the study
☒	☐ ChIP-seq
☐	☒ Flow cytometry
☒	☐ MRI-based neuroimaging

Antibodies

Antibodies used: Cell Signaling Technologies Myc-Tag (9B11) Mouse mAb (Alexa Fluor® 647 Conjugate)

Validation: Monoclonal antibody was validated by manufacturer to specifically target human c-Myc residues 410-419 (EQKLISEEDL). Manufacturer-provided validation includes specific staining of Myc-tagged proteins but not cell extracts via Western blot. We performed independent validation in yeast for specific labeling of Myc-tag expressing yeast.

Eukaryotic cell lines

Policy information about cell lines

Cell line source(s): Hi5 and SF9 cells were originally from ThermoFisher

Authentication: Cell lines were used for protein expression and were not authenticated

Mycoplasma contamination: Cell lines were used for protein expression and were not tested for mycoplasma

Commonly misidentified lines (See ICLAC register): No commonly misidentified lines were used in this study.

Flow Cytometry

Plots

Confirm that:

☒ The axis labels state the marker and fluorochrome used (e.g. CD4-FITC).

☒ The axis scales are clearly visible. Include numbers along axes only for bottom left plot of group (a 'group' is an analysis of identical markers).

☒ All plots are contour plots with outliers or pseudocolor plots.

☒ A numerical value for number of cells or percentage (with statistics) is provided.

Methodology

Sample preparation: Yeast were twice washed into PBS 7.4 containing 0.5% BSA and 1 mM EDTA (FACS Buffer), stained with fluorescent antibody for 30 minutes, and washed twice in FACS buffer for analysis

Instrument: BD Biosciences Accuri C6

Software: BD Biosciences CSampler Software

Cell population abundance: Yeast were not sorted, but rather, selected by Alexafluor647 magnetic beads (Miltenyi Biotec) for labeling with myc-tag Alexafluor647 antibody (described above)

Gating strategy: Yeast were gated based upon their diagonal log-log FSC-A vs. SSC-A profile (Supplemental Figure 1A). Myc positive population was defined as all yeast within this gate that had a higher Alexafluor-647 fluorescent signal than untransduced yeast.

☒ Tick this box to confirm that a figure exemplifying the gating strategy is provided in the Supplementary Information.
