# Supplementary material for: Repertoire-scale determination of class II MHC peptide binding via yeast display improves antigen prediction
Source: Nat Commun. 2020 Sep 4;11:4414. doi: 10.1038/s41467-020-18204-2 (PMC7473865; doi:10.1038/s41467-020-18204-2)
Supplement: Supplementary file 3 — Description of Additional Supplementary Files [file 41467_2020_18204_MOESM3_ESM.pdf]

Description of Additional Supplementary Files

File Name: Supplementary Data 1
Description: Contains peptide sequences from each round of yeast display selections for each experiment in the manuscript, and the sequences used for reference datasets from mass spectroscopy experiments.

File Name: Supplementary Data 2
Description: Contains frequency and Log_2FC values for peptide selection data used to generate heat maps.
